# Improved preanalytical workflow for pancreatic tissue lipidomics: insights into lipid stability and polar lipid recovery

**DOI:** 10.1016/j.jlr.2025.100968

**Published:** 2025-12-26

**Authors:** Karol Parchem, Malena Manzi, Robert Jirásko, Ondřej Peterka, Zuzana Lásko, Ondřej Kuda, Michal Holčapek

**Affiliations:** 1 Department of Analytical Chemistry, Faculty of Chemical Technology, University of Pardubice, Pardubice, Czech Republic; 2 Institute of Physiology, Czech Academy of Sciences, Prague, Czech Republic

**Keywords:** tissue lipidomics, pancreas, sample preparation, lipid fractionation, supercritical fluid chromatography, MS

## Abstract

Tissue lipidomics is a rapidly advancing field in clinical and biomedical research that provides crucial information on the lipid-driven molecular mechanisms underlying physiological and pathological conditions. However, accurate MS-based analysis requires careful preanalytical handling due to the metabolic activity of tissue and analyte heterogeneity. Here, we introduce a robust tissue processing workflow with the pancreas as a model of a highly metabolically active organ. First, we evaluate lipid stability in porcine pancreatic tissue stored on ice, observing significant lysophospholipid formation after 60–120 min. Then, we compare sample handling using ice versus liquid nitrogen for both porcine and mouse pancreatic tissues, illustrating that processing temperature affects low-abundant lipid class levels, with liquid nitrogen providing better preservation. To enhance polar lipidome analysis, we optimize a hexane-methanol liquid-liquid extraction protocol and find that the addition of 2% (v/v) water to methanol yields the most effective recovery and reproducibility. Finally, the workflow is applied to mouse pancreatic tissue samples, enabling the identification of 209 polar lipid species across 10 classes, with 124 species quantified. Among these, hexosylceramides show clear sex-specific variation.

## Graphical abstract

**Figure:**
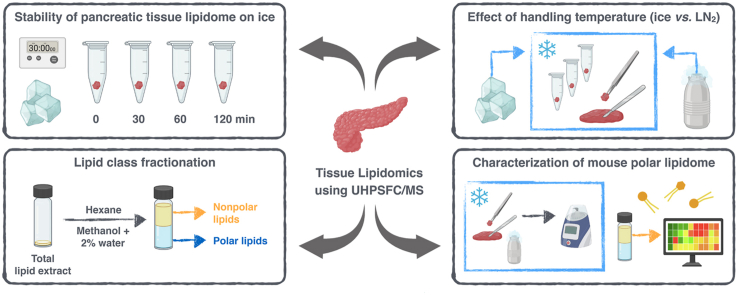

Lipids are a diverse group of biomolecules that play various important roles in biological systems, such as energy storage, membrane formation, endogenous signaling, etc. (1). Importantly, lipid composition and its molecular networks and metabolism were found to exhibit organ-, tissue-, cell-, and even organelle-type specificity (2, 3, 4). On the other hand, previous studies have demonstrated that the blood and tissue lipidomes appear to be somewhat modifiable by external and internal stimuli, with some organs having greater or lower flexibility of the lipid profile than others (4). Among these factors, diet (e.g., PUFA supplementation (5, 6, 7)), physical activity (8), age (9), sex, or genotype (4) can be distinguished. Moreover, the dysregulation of lipid metabolism and related changes in circulating and/or tissue-specific lipids have been linked to the development and progression of multiple serious disorders, including nonalcoholic fatty liver disease (10), type 2 diabetes (11), and various types of cancer, such as colorectal (12), kidney (13), or lung (14, 15).

Plasma is one of the most studied types of biological samples in lipidomic research. This resulted in the development of protocols for untargeted and targeted plasma lipid analysis using various methods (16, 17, 18), workflow harmonization across laboratories (19), and the establishment of standardized guidelines (20). These efforts have resulted in the identification of up to 600 lipid species in plasma samples (17, 21). The lipidomic characterization of tissue samples, unlike plasma analysis, provides specific insights into the molecular mechanisms underlying physiological and pathological conditions at the organ or tissue level. In addition, this approach can aim to correlate tissue diagnostic biomarkers, whose determination generally requires an invasive sampling procedure, with the lipid profile identified in the circulatory system or urine (22). However, in contrast to biofluids, tissue samples are highly metabolically active (23) and have a heterogeneous distribution of analytes (24). Therefore, the lipidomic analysis of tissue samples is more challenging and difficult to perform in a high-throughput manner. This is related to more complicated handling of tissue samples, including sampling, aliquoting, and homogenization (24). Moreover, the analysis of different tissue samples (e.g., from various organs or anatomical parts) requires tissue-specific optimization steps, such as the adjustment of the internal standard (IS) mixture composition or total lipid extract purification/fractionation due to a wide concentration range of endogenous lipid classes (25).

The organ most commonly analyzed in animal studies is the liver, and several studies on preanalytical optimization have been performed (26, 27, 28). Other analyzed organs or tissues are the brain, kidney, lung, muscle, or adipose tissue (4, 29). However, little attention has been paid to the pancreas, and in multiorgan lipidomic studies, it is often neglected (4, 29, 30, 31). The pancreas is an important organ regulating the body’s energy homeostasis and plays a dual role in the organism: endocrine (production of hormones, e.g., insulin) and exocrine (production of digestive enzymes) (32). The pancreas may be affected by numerous serious disorders, such as diabetes mellitus, pancreatic steatosis, acute and chronic pancreatitis, or pancreatic cancers, including pancreatic ductal adenocarcinoma (33). To understand the molecular mechanisms involved in the development of these diseases, the lipidomic analysis of pancreatic tissue is increasingly employed (34, 35). Out of neutral lipids, whose abnormal accumulation in pancreatic tissue can be observed in the course of pancreatic disorders (33), the dysregulation of polar lipid classes is commonly associated with their development (35).

The lipidomic characterization of pancreatic tissue can contribute to the discovery of new biomarkers of the disorders mentioned. However, accurate lipidomic analysis of pancreatic tissue requires a careful evaluation of multiple preanalytical factors, due to the scarcity of previous studies and the expected high metabolic activity of the tissue. The pancreas is rich in digestive lipolytic enzymes, such as pancreatic lipase, pancreatic phospholipase A_2_ (PLA_2_), carboxyl ester lipase, or galactolipase (36). As a result of tissue homogenization, zymogen forms of these enzymes can be liberated and activated by autoproteolysis (37). In addition, pancreatic tissue is characterized by relatively high heterogeneity, which can hinder the collection of representative samples. In the liver, fat is mainly accumulated in intracellular lipid droplets of hepatocytes (38). However, in the case of pancreas, apart from lipid accumulation in lipid droplets (39), fat is stored in adipocytes that invade the parenchyma (40). Therefore, special consideration must be given to the collection of representative samples in terms of size, location, and number of replicates (41).

Considering the current challenges in the field of organ-specific tissue lipidomics, our objective was to develop a preanalytical workflow for the processing of pancreatic tissue as an example of a metabolically active sample type. First, we assessed the stability of tissue lipidome during storage of samples on ice up to 120 min using porcine pancreas as a model. Next, we compared the effect of temperature (ice vs. liquid nitrogen) during tissue/organ handling on changes in lipid profile using porcine and mouse pancreatic samples. These steps were intended to determine the temperature conditions that minimize the enzymatic degradation of lipids in pancreatic tissues. On the other hand, the crude extracts of tissue samples contain lipid classes with a wide range of hydrophobicity and concentrations. The direct analysis of these types of extracts can complicate the analysis and increase the risk of chromatographic system and mass spectrometer contamination. Therefore, we optimized the liquid-liquid extraction (LLE) of the total lipid extract to separate nonpolar and polar lipid classes. Finally, we applied the optimized workflow for qualitative and quantitative analyses of polar lipidomes of female and male mouse pancreas, employing ultrahigh performance supercritical fluid chromatography (UHPSFC) coupled to high-resolution MS analysis.

## Materials and methods

### Chemicals and solvents

Chloroform (LC grade) was purchased from Merck (Darmstadt, Germany). Hexane (LC grade), methanol (MeOH), and water (both LC/MS grade) were purchased from Honeywell Riedel-de Haën (Seelze, Germany), as were ammonium carbonate (reagent grade) and ammonium acetate (≥99.99%). Carbon dioxide (99.995% purity) was obtained from Messer Group (Bad Soden, Germany). Deionized water was prepared using a Milli-Q Reference purification system (Millipore, Molsheim, France). The lipid standards (Table 1) were obtained from Avanti Polar Lipids (Alabaster, AL) and Nu-Chek Prep (Elysian, MN). Lipid shorthand nomenclature follows the published recommendations (42).

**Table 1:** Composition of IS mix used for the quantification of individual lipid species within each lipid class present in mouse pancreatic tissue

Lipid class (Abbreviation)	Primary IS	Concentration (nmol/mL of IS mix)	Concentration (nmol/mg of tissue)	Secondary IS	Concentration (nmol/ml of IS mix)	Concentration (nmol/mg of tissue)
Cholesteryl ester^a^ (CE)	CE 16:0 d7	64.1	0.26	CE 17:0	63.4	0.25
Ceramie (Cer)	Cer C18 d7	5.0	0.020	Cer 35:1	5.0	0.020
Cholesterol^a^ (Chol)	Chol d7	914.4	3.77	—	—	—
Diacylglycerol^a^ (DG)	DG 33:1 d7	2.5	0.010	DG 36:2 d5	2.6	0.010
Hexosylceramide (HexCer)	GlcCer 36:1 d5	3.72	0.015	GlcCer 30:1	3.73	0.015
Lysophosphatidylcholine (LPC)	LPC 18:1 d7	43.5	0.17	LPC 13:0	48.5	0.19
Lysophosphatidylethanolamine (LPE)	LPE 18:1 d7	7.5	0.030	LPE 14:0	7.5	0.030
Monoacylglycerol^a^ (MG)	MG 18:1 d7	14.3	0.060	MG 19:1^b^	15.1	0.057
Phosphatidylcholine (PC)	PC 33:1 d7	562.2	2.25	PC 28:0	562.2	2.25
Ether phosphatidylcholine (PC O-); Phosphatidylcholine plasmalogen (PC-P)	PC P-36:1 d9	50.0	0.20	—	—	—
Phosphatidylethanolamine (PE)	PE 33:1 d7	224.9	0.90	PE 28:0	224.9	0.90
Ether phosphatidylethanolamine (PE-O); Phosphatidylethanolamine plasmalogen (PE-P)	PE P-36:1 d9	219.2	0.88	—	—	—
Phosphatidylglycerol (PG)	PG 33:1 d7	8.7	0.035	PG 28:0	9.8	0.039
Phosphatidylinositol (PI)	PI 33:1 d7	42.5	0.17	PI 33:1	42.9	0.17
Phosphatidylserine (PS)	PS 33:1 d7	85.7	0.34	PS 28:0	85.4	0.34
Sphingomyelin (SM)	SM 36:2 d9	65.0	0.26	SM 30:1	64.9	0.26
Triacylglycerol^a^ (TG)	TG 48:1 d7	164.0	0.66	TG 57:3^b^	142.3	0.57

a Not for quantitative purpose.

b Purchased from Nu-Chek. All other standards were obtained from Avanti Polar Lipids.

### Animal pancreatic tissue samples

The porcine pancreas was obtained from a local butcher after slaughter, immediately frozen at −20 °C, transported on dry ice, and stored at −80 °C in ∼1 g aliquots. Random sampling was used to cover different regions of the organ. C57BL/6J male and female mice (n = 3 per group, age: 18–19 weeks) were euthanized and dissected. Pancreatic tissue was snap-frozen in liquid nitrogen, shipped in 1.5 ml Eppendorf tubes on dry ice, and kept at −80 °C until processing. The guidelines for the care and use of laboratory animals of the Institute of Physiology of the Czech Academy of Sciences were followed, and the experiments were approved by the Committee for Animal Protection of the Czech Academy of Sciences (protocol nos.: 172/2009 and 81/2016).

### Study design

**Fig. 1:**
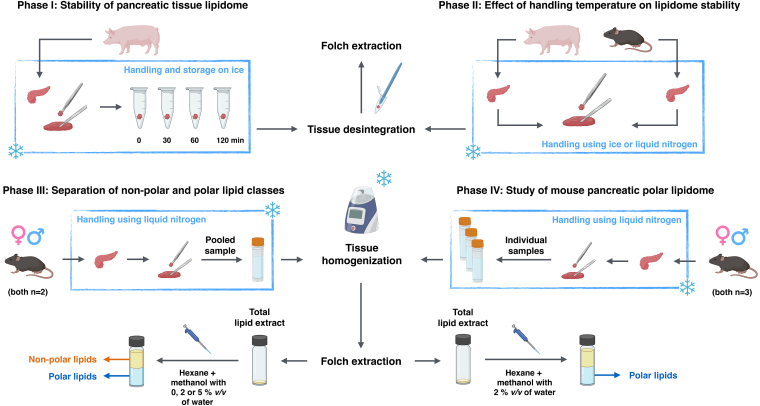
Study design. Created with BioRender.com.

The study was designed in four consecutive phases (Fig. 1). In phase I, porcine pancreatic tissue (10–20 mg) was stored on ice for 0, 30, 60, or 120 min to assess lipidome stability. To minimize technical variability, tissue samples were sequentially removed from the freezer at −80°C at defined time points, allowing manual homogenization and subsequent extraction of lipids from all samples at the same final time point. Phase II evaluated the impact of temperature during tissue aliquoting, using mouse and porcine tissue samples handled either on ice or in liquid nitrogen. The samples obtained (10–20 mg) were processed immediately or stored at −80°C prior to manual homogenization and lipid extraction. Upon completion of phase I and II, the time-consuming manual tissue disintegration approach was replaced with high-throughput bead-based homogenization, allowing for increased efficiency and precise control over homogenization time and intensity. In phase III, LLE of polar and nonpolar lipids using hexane and MeOH (with or without added water) was optimized. For this purpose, samples (10–20 mg) from two male and two female mice were pooled and bead-homogenized, followed by lipid extraction and fractionation. Phase IV applied the full optimized workflow to the polar lipidomic profiling of pancreatic tissue samples (20 mg) from three female and three male mice using bead-based homogenization, extraction, fractionation, and UHPSFC/MS analysis.

### Tissue sample handling with ice

A glass Petri dish was washed with chloroform/MeOH (1:1 v/v), dried, and chilled on ice for ∼10 min. Deep-frozen (−80°C) porcine tissue sample (∼1 g aliquot) or mouse pancreas was transferred onto a dish, left to partially thaw for approximately 2 min, cut using a scalpel and tweezers, and then transferred to preweighed 1.5 ml Eppendorf tubes. Tubes were reweighed, then either processed immediately on ice or refrozen at −80°C.

### Tissue sample handling with liquid nitrogen

An aluminum block (15 × 12 × 5 cm) in a polystyrene box was flooded with liquid nitrogen and cooled for 15 min. Deep-frozen (−80°C) porcine tissue sample (∼1 g aliquot) or mouse pancreas was transferred to an aluminum foil placed on the block and cut/crushed. The pieces were transferred to preweighed 1.5 ml tubes, reweighed, placed in liquid nitrogen, and processed immediately or stored at −80°C.

### Manual tissue disintegration with a pestle

Ice-cold MeOH (150 μl) was added to the 1.5 ml polypropylene tube containing 10–20 mg of pancreatic tissue. If frozen, the samples were softened by storage for 30 s at room temperature. Disruption was performed with a disposable pestle (∼30 s), which was rinsed with another 150 μl MeOH. The resulting homogenate (300 μl total) was transferred to 4 ml glass vial containing 2 ml chloroform with 20 μl IS mix. Polypropylene tubes were rinsed twice with 350 μl MeOH each time, and rinsates were pooled into the same glass vial.

### Bead-based tissue homogenization

Frozen pancreatic tissue (10–20 mg) was transferred to 2 ml reinforced tubes with 2.8 mm ceramic beads (CK28-R kit; Bertin Technologies). Tubes were prefilled with ice-cold MeOH to achieve the concentration of 40 mg/ml (tissue to solvent ratio: 1:25 w/v). Homogenization was performed using the Precellys Evolution with Cryolys system (Bertin Technologies) at 4 °C, 8,000 rpm, 3 × 20 s cycles, with 30 s intervals. An aliquot (75 μl corresponding to 3 mg of tissue) was transferred to a 4 ml glass vials containing 2 ml chloroform and 925 μl MeOH with 20 μl IS mix.

### Lipid extraction

Lipid extraction was carried out according to Folch *et al*. (43), with some modifications. Disintegrated or bead-based homogenized tissue samples, after transfer to the chloroform/MeOH mixture, were vortexed (10 s), stored on ice for 30 min, and subsequently sonicated (FB15050 S-series ultrasonic bath; Fisherbrand) for 15 min at ambient temperature. Aqueous solution of 250 mM ammonium carbonate (0.6 ml) was added, the samples were vortexed, shaken (KS 130 Basic orbital shaker, IKA, 750 rpm, 5 min), and centrifuged (EBA 20 centrifuge, Hettich, 5,000 rpm, 5 min). The organic (bottom) phase was transferred to 8 ml glass vials. The aqueous phase was re-extracted with 2 ml of chloroform and centrifuged again. Organic phases were combined, and solvents were evaporated under nitrogen at 30°C. Lipid residues were stored at −80°C.

### Lipid fractionation

For the separation of nonpolar and polar lipids, LLE with hexane and MeOH was used. To achieve the best recovery of polar lipids, MeOH with the addition of 0, 2, or 5% (v/v) water was evaluated. In brief, lipid residues were dissolved in 2 ml hexane and 1 ml of nonaqueous or aqueous MeOH, samples were vortexed three times for 30 s each, and then centrifuged (5,000 rpm, 5 min). The upper hexane-rich layer was collected into a new vial, and solvents from both phases were evaporated under nitrogen (30°C) and stored at −80°C.

### UHPSFC/MS analysis

Lipid extracts were reconstituted in 500 μl chloroform/MeOH (1:1 v/v) and diluted 10 to 200 times depending on the fraction type (total, nonpolar, or polar). Lipid classes were separated using a previously published UHPSFC/MS method (16, 44) with an Acquity UPC2 system (Waters) and Viridis BEH column (100 × 3 mm, 1.7 μm, Waters). The mobile phases consisted of supercritical CO_2_ (A) and MeOH containing 30 mM ammonium acetate with 1% (v/v) water (B). The mobile phase gradient was 0 min—1% B; 1.5 min—16% B; 4 min—51% B; 7 min—51% B; 7.51 min—1% B; 8 min total run. The following parameters were used: flow rate, 1.9 ml/min; column temp, 60°C; automatic back-pressure regulator, 1800 psi; and injection volume, 1 μl. The system was coupled to a Synapt G2-Si QTOF mass spectrometer (Waters) with a commercial interface, enabling postcolumn make-up flow and pressure control. The make-up solvent (same as phase B) was infused at 0.25 ml/min. The following MS conditions were applied: capillary voltage, +3.0 kV for positive ion mode and −2.5 kV for negative ion mode; *m/z* 150–1,200; sample cone, 20 V; source offset, 90 V; source temperature, 150°C; desolvation temperature, 500°C; cone gas flow, 50 L/min; desolvation gas flow, 1000 L/min; and nebulizer pressure, 4 bar. Data were acquired in continuum mode (0.5 s scan time), including leucine enkephalin as lock mass.

### Data processing

The raw MS data were processed using MassLynx 4.1 software (Waters). Files were noise-reduced (Water Compression Tool), lock-mass corrected, and converted to centroid mode (Accurate Mass Measure Tool). Chromatograms of early, mid, and late sequence samples were reviewed to define scan ranges for each lipid class. MarkerLynx methods (20 mDa mass window, intensity threshold 3,000) were applied across samples. Exported .txt files were processed using in-house LipidQuant 2.1 software (45), enabling the lipid species identification with 10 ppm mass tolerance, type I and II isotopic correction, and quantification. However, all detected features were manually checked, and only manually confirmed identifications were used for data evaluation. Moreover, retention times of individual lipid classes were confirmed by ISs. Two ISs were used per lipid class (at least one deuterated), except for cholesterol (Chol), which had only one deuterated IS. Individual lipid species within each lipid class were quantified using a one-point calibration approach with the corresponding primary (deuterated) IS for each lipid class. The ratio of the peak area of each lipid species (after type I and II isotopic corrections) to that of the deuterated IS was calculated and multiplied by the amount of the IS added to the sample during extraction. The concentrations of ISs in the in-house-developed IS mix were selected so that the ratio of the most abundant lipid species in the sample to the deuterated IS within each individual lipid class ranged between 2:1 and 3:1. The secondary (typically nondeuterated) IS, at a concentration similar to that of the deuterated IS, was used to back-calculate its concentration based on the primary IS, and subsequently, to estimate the quantification error (46). Lipid species concentrations were expressed as nanomoles/milligram of wet tissue.

Quantitative data were filtered in three steps: *i*) blank correction—lipids with blank signals exceeding 20% of the quality control (QC) mean intensity were excluded; *ii*) robustness filter—only lipids detected in all QC samples with a coefficient of variation of concentrations below 20% were retained; and *iii*) prevalence filter—lipids detected in minimum 80% of samples were kept. Missing values were imputed as 80% of the minimum concentration within a given sample group.

### Statistical analysis and visualization

Data were expressed as mean ± SD of three replicates, unless otherwise stated. Statistical analysis was performed using ANOVA or an unpaired *t*-test with a significance threshold set at *P* ≤ 0.05. Detailed information about the tests used for each dataset is provided in the figure and table captions. Both statistical analysis and graphical visualizations were performed using Prism 10.3.0 software (GraphPad Software). Principal component analysis (PCA) and volcano plots were performed using MetaboAnalyst 6.0 (https://www.metaboanalyst.ca/).

Detailed information on sample overview and preanalytics, lipid extraction, analytical platform, lipid identification and quantification, and QC is available in the Reporting Checklist (Supplementary Data 1) developed by the Lipidomics Standard Initiative (https://lipidomicstandards.org/) (47).

## Results

### Stability of pancreatic lipidome during tissue sample storage on ice (phase I)

In phase I, porcine pancreatic tissue was used because a relatively large amount of biological material can easily be obtained for a preliminary assessment of tissue stability during sample handling. Our initial experiments on porcine pancreatic samples (collected from different regions of one organ) handled at ambient temperature revealed significant variations between processed samples, particularly in lysophospholipid (lyso-PL) levels (Supplemental Fig. S1). These observations prompted us to assess tissue stability during storage under reduced temperature conditions. For this purpose, porcine pancreatic tissue samples were stored on ice for 0 (control), 30, 60, or 120 min. Immediately after each storage time point, samples were homogenized, and lipids were extracted. Lipids were quantified by UHPSFC/MS using the lipid class separation approach.

**Fig. 2:**
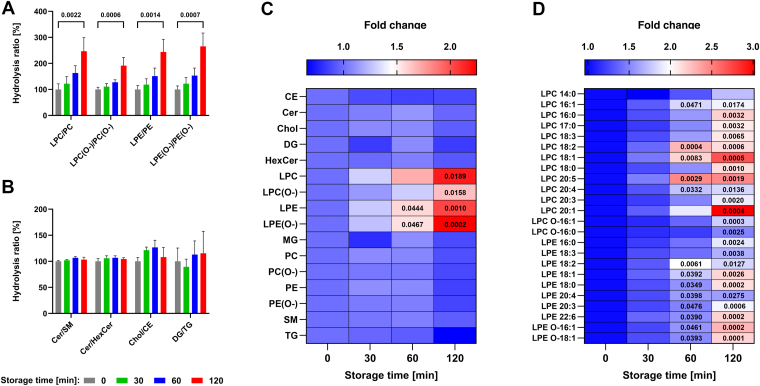
Stability of the porcine pancreatic lipidome during tissue storage on ice. A: Hydrolysis ratios of individual precursor/degradation lipid classes after 0 (control, set to 100%), 30, 60, and 120 min of storage. B: Temporal changes in other investigated lipid classes. C: Heatmap depicting the alterations in total peak areas of individual lipid classes during sample storage. D: Heatmap illustrating changes in individual LPC and LPE species over time. Data represent means ± SD from three independent experiments. Statistical significance was determined using one-way ANOVA with Dunnett’s post hoc test (*P* ≤ 0.05).

To evaluate the lipidome stability, the hydrolysis ratios were calculated as the total peak area of species belonging to lipid classes representing degradation products relative to the total area of species of the corresponding precursor lipid classes (28). The ratios for individual degradation/precursor lipid class pairs at the time of 0 min were set to 100%. For all glycerophospholipid (GPL) pairs, namely lysophosphatidylcholine (LPC)/phosphatidylcholine (PC), LPC(O-)/PC(O-), lysophosphatidylethanolamine(LPE)/phosphatidylethanolamine (PE), and LPE(O-)/PE(O-), a statistically significant increase (*P* ≤ 0.05) was observed between the control (0 min) and 120 min of sample storage (Fig. 2A). However, all GPL-related hydrolysis ratios showed an increasing trend throughout the storage period. For the remaining lipid classes, no significant effect of tissue storage time on their quantities was found (Fig. 2B). Moreover, the highest variations in the degradation/precursor lipid class ratios were observed for the diacylglycerol (DG)/triacylglycerol (TG) and Chol/cholesteryl ester (CE) pairs, with coefficient of variation values reaching up to 36% (Fig. 2B). However, the hydrolysis ratios do not accurately reflect the changes observed at the precursor and product class levels. Therefore, the relative changes in the total peak areas of individual lipid classes were additionally illustrated as a heatmap. These data revealed a statistically significant increase in the LPE and LPE(O-) classes already after 60 min of storage (Fig. 2C). Whereas for LPC and LPC(O-), a statistical difference was observed after 120 min, which is consistent with the results obtained based on the hydrolysis ratios. Conversely, no statistically significant decrease in the relative total peak area of precursor GPLs was detected across all storage times (Fig. 2C).

The obtained data indicated that storage of porcine pancreatic tissue samples on ice leads to the formation of lyso-PLs. Importantly, all identified lyso-PL species, except LPC 14:0, exhibited a statistically significant increase (*P* ≤ 0.05) in relative peak areas between the control and 120 min of storage, whereas some species already showed statistically significant changes after 60 min (Fig. 2D). The highest formation rates over the entire storage period were observed for LPC 18:2, LPC 18:1, and LPC 20:5 species, whereas LPC 20:1 exhibited the highest mean peak area at the storage endpoint. In contrast, LPC O-16:0, LPE 20:4, and LPE 18:3 exhibited the slowest increase in peak areas during the storage period. Meanwhile, statistical analysis of all precursor GPLs revealed a significant decrease (*P* ≤ 0.05) in relative peak areas between the control and 120 min of storage on ice for only a few species, namely PC 38:6, PC 40:9, PE 36:3, and PE O-33:3 (Supplemental Table S1).

### Effect of handling temperature on porcine and mouse pancreatic tissue lipidome (phase II)

**Fig. 3:**
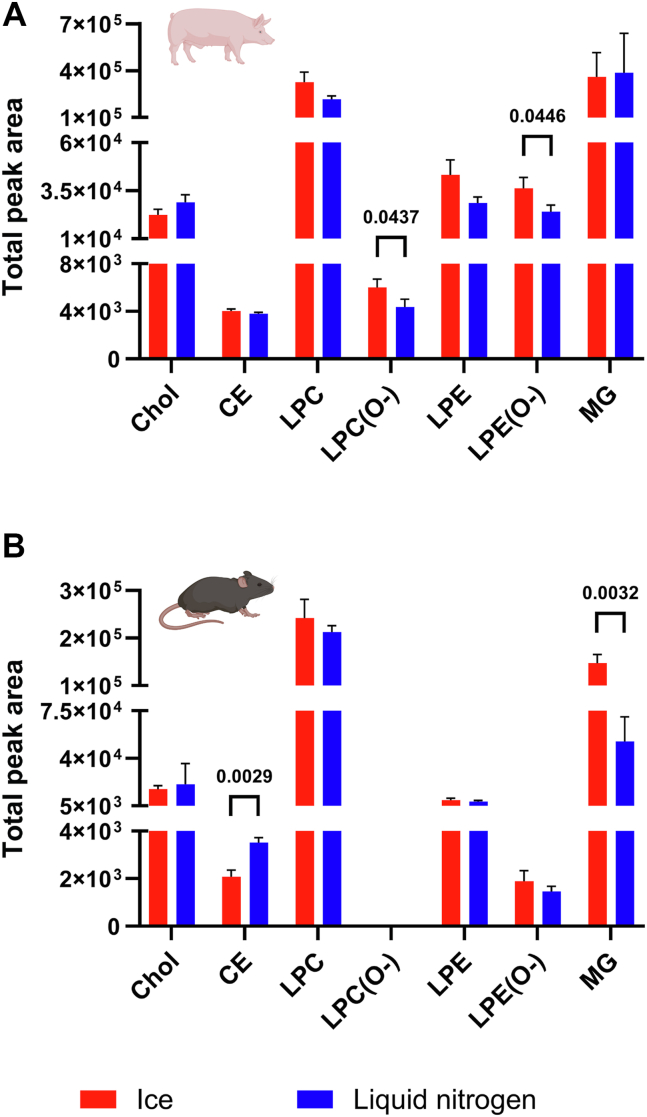
Effect of handling temperature (ice vs. liquid nitrogen) on alterations in total peak areas of low-abundance lipid classes in (A) porcine and (B) mouse tissue samples. Data represent means ± SD from three independent experiments. Statistical significance was determined using an unpaired *t*-test with Welch’s correction for each lipid class (*P* ≤ 0.05).

Phase II aimed to compare the effect of temperature conditions during tissue handling, including sample aliquoting and intermediate sample storage (e.g., prior to the addition of homogenization solvent). For this purpose, porcine and mouse pancreatic tissue samples were handled using ice or liquid nitrogen. After aliquoting, the samples were immediately processed or stored at −80°C prior to homogenization, and the lipids were extracted and then analyzed using UHPSFC/MS. Comparison of total peak areas of individual lipid classes between samples handled on ice and in liquid nitrogen revealed statistically significant differences (*P* ≤ 0.05) for a few lipid classes, which varied between animal species tested. In porcine tissue, significantly lower levels of LPC(O-) and LPE(O-) were observed in samples processed in liquid nitrogen (Fig. 3A). In contrast, mouse samples processed under the same conditions showed significantly lower levels of monoacylglycerols (MGs) and higher CE levels compared with ice-handled samples (Fig. 3B). It should be noted, however, that significant differences in lipidome composition were observed between porcine and mouse pancreatic tissue, which in itself may make it somewhat difficult to compare the effect of processing temperature across animal species. For example, mouse tissue was characterized by low levels or the absence of ether lyso-PL species, even in samples processed using liquid nitrogen (Supplemental Table S3). In contrast, in pancreatic tissue, the higher abundance of these lipids enabled the detection of statistically significant differences between samples processed on ice and those processed using liquid nitrogen.

**Fig. 4:**
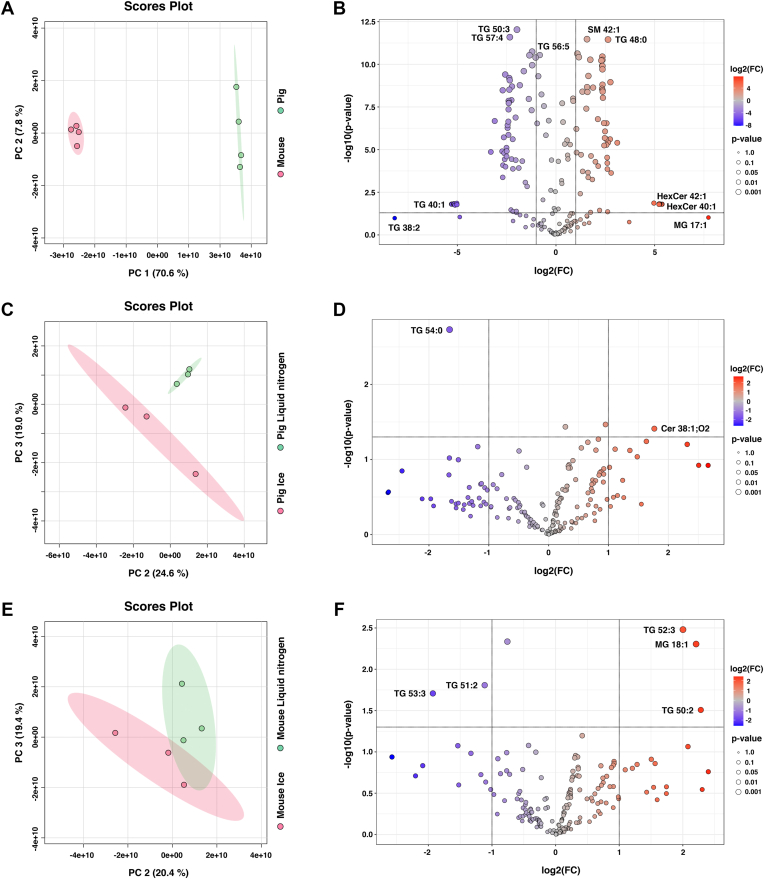
Differences in the lipidome composition between porcine and mouse pancreatic tissues and the effect of sample handling temperature on lipid stability. A: Two-dimensional (2D) PCA score plots for porcine and mouse pancreatic tissue total lipid extracts (obtained by pooling all individual extracts per animal). B: Volcano plots highlighting significantly different lipid species between porcine and mouse pancreatic tissue total lipid extracts (direction of comparison: pig/mouse). C and E: PCA score plots for porcine and mouse pancreatic tissue total lipid extracts processed using ice and liquid nitrogen, respectively. D and F: Corresponding volcano plots showing significantly different lipid species between samples handled using ice and liquid nitrogen for porcine and mouse pancreatic tissues. Analyses and visualizations were performed using MetaboAnalyst 6.0. The vertical lines indicate a fold change (FC) threshold of 2, whereas the horizontal line represents a statistical significance level of 0.05, as determined by the *t* test. LN2, liquid nitrogen 2.

Subsequently, the obtained lipidomic data were evaluated using PCA. When applied to porcine and mouse total lipid extracts (prepared by pooling individual extracts per animal and used as QC samples during sequence analysis of each organ), the analysis revealed two well-separated groups corresponding to the respective animal species (Fig. 4A). Among 208 identified lipid species, 149 were found in both porcine and mouse pancreatic tissue lipid extracts. Moreover, 120 species showed statistically significant differences (*P* ≤ 0.05) and exhibited a fold change greater than 2 (Fig. 4B). Among species with the lowest *P* values between porcine and mouse pancreatic tissues were SM 42:1, TG 48:0, and TG 50:3. Moreover, the lipidomic analysis of total lipid extracts did not reveal the presence of hexosylceramide (HexCer) species, specifically HexCer 42:1, HexCer 40:1, and HexCer 38:1, in mouse samples, although they were detected in porcine tissue.

The PCA of porcine pancreatic tissue samples processed on ice and in liquid nitrogen (both n = 3) showed that the 2D score plot did not reveal sample clustering related to different processing strategies (Supplemental Fig. S2A). However, the 3D score plot revealed that the group separation became apparent in the 2D plane defined by principal component 2 and principal component 3 (Fig. 4C). The top five features with the highest loadings on principal component 2 and principal component 3 were PC 38:1, PE O-36:5, TG 52:4, LPE O-18:1, and PC 32:2, as illustrated in the scores and loadings plot (biplot) (Supllemental Fig. S2E). The volcano plot revealed that only two lipid species, namely TG 54:0 and ceramide (Cer) 38:1;O2, were statistically significantly different (*P* ≤ 0.05) and were characterized by the fold change greater than 2 (Fig. 4D). For mouse pancreatic tissue samples processed using ice and liquid nitrogen, the 2D score plot including principal component 1 and principal component 2 also showed no group separation based on lipidome composition (Supplemental Fig. S3C), indicating limited variance between groups. Similarly to porcine samples, the 3D score plot was analyzed for mouse tissue, revealing partial separation between groups representing processing with different strategies in the 2D plane defined by principal component 2 and principal component 3 (Fig. 4E). Here, the top five features with the highest loadings on principal component 2 and principal component 3 were DG 32:1, TG 54:4, DG 36:3, TG 50:2, and TG 47:0 (Supplemental Fig. S2F). In the volcano plot, the following lipid species were found to be significantly different (*P* ≤ 0.05) with a fold change greater than 2: TG 52:3, MG 18:1, TG 51:2, TG 53:3, and TG 50:2 (Fig. 4F).

### Fractionation of polar and nonpolar lipids by LLE (phase III)

Total lipid extracts isolated from animal tissue samples typically contain numerous lipid classes with different polarities and a wide range of concentrations, making the comprehensive lipid analysis in a single LC/MS run challenging. Initially, we observed an intense signal for the TG class, which posed a limitation in the detection and quantification of less abundant but biologically relevant classes, such as LPE, which are known to be involved in pancreatic diseases (35). To address this issue, in phase III of our development, we implemented an inexpensive and time-efficient LLE of total lipid extracts, enabling the effective separation of polar and nonpolar lipid classes. The solvent system used consisted of hexane and MeOH (2:1 v/v), whereas the addition of water to MeOH (0, 2, or 5% v/v) was optimized to promote the selective distribution of polar and nonpolar classes between phases. After the separation, the solvents from both phases were evaporated, and samples were prepared for UHPSFC/MS analysis in both polarity modes for the MeOH-rich phase and in the positive ion mode for the hexane-rich phase.

**Fig. 5:**
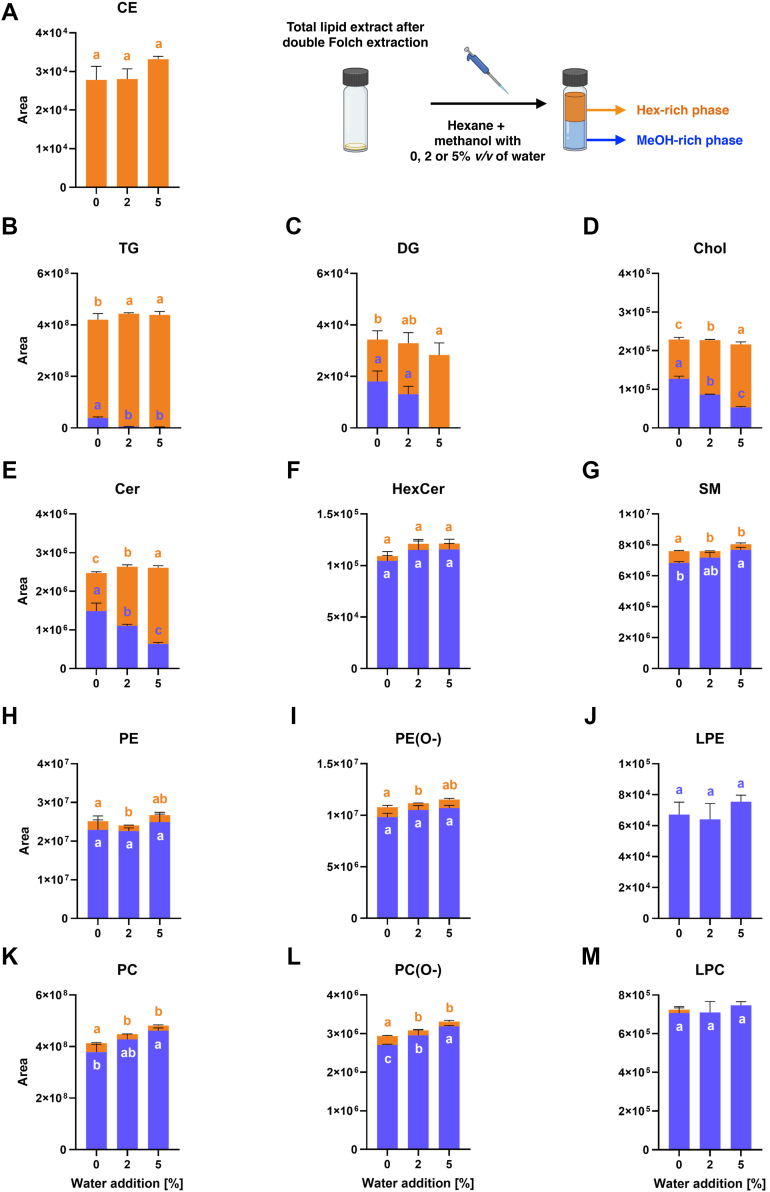
Effect of water content in methanol used during liquid-liquid fractionation of total lipid extract from mouse pancreatic tissue on lipid class partitioning between MeOH- and hexane-rich phases. Data represent the total peak areas of all identified lipid species within individual lipid classes: (A) CE; (B) TG; (C) DG; (D) Chol; (E) Cer; (F) HexCer; (G) SM; (H) PE; (I) ether PEs (PE(O-)); (J) LPE; (K) PC; (L) ether PC (PC(O-)); and (M) LPCs. Results are presented as means ± SD from three independent analyses. Different letters indicate statistically significant differences examined using one-way ANOVA with Tukey’s post hoc test (*P* ≤ 0.05).

The most hydrophobic lipid classes identified in the mouse pancreatic tissue lipid extract obtained by Folch extraction, namely CE and TG, were found exclusively or predominantly in the hexane-rich phase (Fig. 5A, B). Beyond the endogenous lipid species, the partitioning behavior was also evaluated for deuterated IS used during extraction (Supplemental Fig. S3). CE 16:0 d7 and TG 48:1 d7 standards exhibited similar properties and, apart from the hexane-rich phase, were detected in noticeable amounts in the MeOH-rich phase only when anhydrous MeOH was used. This may be attributed to the partial miscibility of hexane in MeOH, which could influence the distribution of standards between two phases. Increasing the water content reduces the miscibility of hexane in the MeOH-rich phase (48), potentially decreasing the solubility of nonpolar lipids in this phase. Moreover, deuterated IS of nonpolar lipid classes, such as CE, TG, DG, and MG, were added in amounts that exceeded the levels of the corresponding endogenous lipid species. Consequently, these ISs were occasionally detected even in fractions where the respective endogenous lipids were not present. This strategy allows for concurrent evaluation of the partitioning behavior of nonpolar classes at concentrations higher than those of endogenous lipids.

For the next neutral lipid class—DG—an increase in the total peak area of identified species in the hexane-rich phase was observed with increasing water content in MeOH. In addition, significant amounts of DG were still detected in the MeOH-rich phase when 0% and 2% (v/v) water was added (Fig. 5C). In both analyzed fractions obtained by LLE, endogenous MG species were not detected. However, the concentration of MG 18:1 d7 IS used during the tissue extraction by the Folch method allowed for its detection; nevertheless, it was found only in the MeOH-rich phase (Supplemental Fig. S3). Chol and Cer classes exhibited similar partitioning behavior (Fig. 5D, E), with a statistically significant decrease in total peak areas of identified lipids in the MeOH-rich phase as the water content in MeOH increased. Moreover, the potential effect of fatty acyl chain structure on the partitioning pattern between MeOH- and hexane-rich phases was evaluated for individual Cer species (Supplemental Fig. S4). In general, all of them exhibited similar behavior, except for Cer 41:2;O2, Cer 36:2;O2, which were the least abundant and likely fell below the detection limit under some fractionation conditions. For HexCer, the total peak area of the identified species was significantly higher in the MeOH-rich phase compared with the hexane-rich one (Fig. 5F).

Similarly to HexCer, no statistically significant effect of the water amount in MeOH used during fractionation was observed for the total peak areas of PE and PE(O-) species in the MeOH-rich phase (Fig. 5H, I). For all tested water contents in MeOH, ethanolamine-containing GPL species were detected in both MeOH- and hexane-rich fractions; however, the molecules exhibited a significantly higher affinity for the MeOH-rich phase. PC and PC(O-) species also followed this trend (Fig. 5K, L). For PC, the statistically significant difference was observed between anhydrous MeOH and the solvent containing 5% (v/v) water, with the higher total peak area detected in the MeOH-rich phase in the latter conditions. In turn for PC(O-) species, the significant differences in the total peak area for the MeOH-rich phase were found between all tested water contents. Another choline-containing lipid class—SM—showed similar partitioning properties to PC, with significantly higher affinity of molecules to the MeOH-rich phase and the highest total peak area of identified species in the hexane phase when no water was added to MeOH (Fig. 5G). Lyso-PL, including LPC and LPE, predominantly partitioned into the MeOH-rich phase (Fig. 5J, M), except for the LPC class, which was also detected in small amounts in the hexane-rich phase when anhydrous MeOH was used.

To minimize the risk of LC system contamination observed during the analysis of the crude pancreatic tissue lipid extract and the hexane-rich phase, only the MeOH-rich phase was analyzed in the negative ion mode. For GPL classes characterized by better ionization efficiency in negative ion mode, namely PG, phosphatidylinositol (PI), and phosphatidylserine (PS), the effect of water content in MeOH on the partitioning behavior was assessed based solely on the analysis of the MeOH-rich phase (Supplemental Fig. S5). However, the addition of water to MeOH had no statistically significant impact on the total peak area of identified species in the MeOH-rich phase for any of the mentioned GPL classes.

### Analysis of mouse pancreatic tissue lipidome (phase IV)

**Fig. 6:**
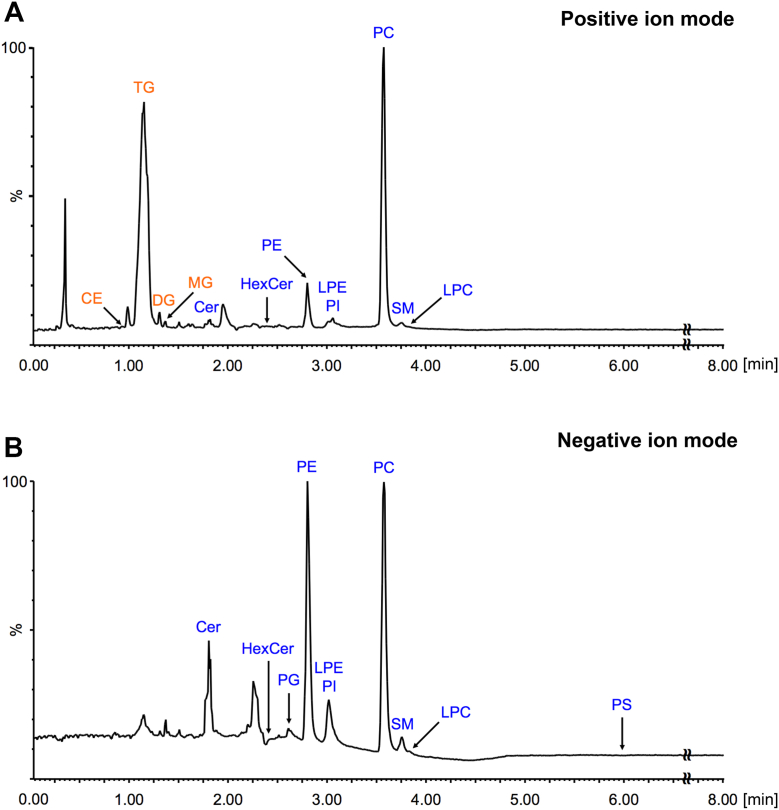
Total ion current chromatograms of crude lipid extracts from mouse pancreatic tissue measured using UHPSFC/MS in (A) positive and (B) negative ion modes.

The last and key phase of the study was the application of the developed workflow for qualitative and quantitative analysis of the mouse pancreatic tissue polar lipidome. For this purpose, tissue samples from three female and three male C57BL/6J mice were used. After lipid extraction using the Folch method followed by polar lipid fractionation using LLE, the samples were analyzed using UHPSFC/MS in positive and negative ion modes (Fig. 6). Individual lipid species were identified based on high-accuracy mass measurement and retention time behavior. Most lipid classes were detected as protonated molecules, except for PI, which was detected as [M + NH_4_]^+^ adducts, and Cer species, which predominantly formed [M + H−H_2_O]^+^ ions. In negative ion mode, LPE, PE, PG, PI, and PS classes were detected as deprotonated molecules, whereas Cer, HexCer, LPC, PC, and SM classes were identified as acetate adducts. A total of 209 polar lipid species were identified in mouse pancreatic tissue, with 126 and 182 species detected in positive and negative ion modes, respectively, including 102 found in both polarities (Supplemental Table S5). In particular, all PG and PS species were identified only in the negative ion mode.

**Fig. 7:**
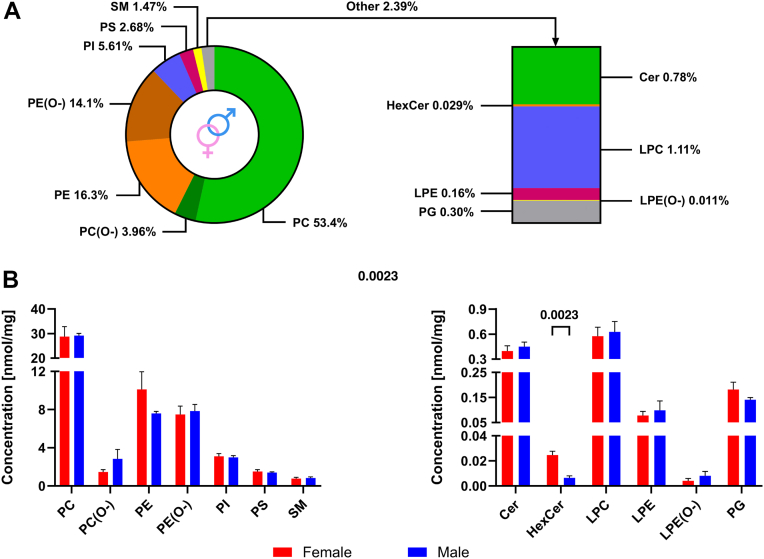
Lipidome composition of mouse pancreatic tissue. A: Percentage composition of polar lipid classes identified in mouse pancreatic tissue samples, including both sexes. B: Concentrations of individual polar lipid classes in pancreatic tissues of female and male mice (n = 3 per group). Results are expressed as means ± SD in nmol/mg of wet tissue. Statistically significant differences between female and male mice were assessed using an unpaired *t*-test with Welch’s correction for each lipid class (*P* ≤ 0.05).

For the quantitative analysis, an approach based on the addition of deuterated IS mix at one concentration point (Table 1), followed by calculation of the concentration using the LipidQuant 2.1 software (45), was employed. Following data curation, 124 of the 209 identified lipid species met all criteria and were quantified. The total concentration of identified polar lipids in mouse pancreatic tissue was 54.18 ± 5.39 nmol/mg of wet tissue, and no statistically significant differences were observed between the sexes. The most abundant lipid classes were PC and PE, which constituted 57.4% and 30.4% of total polar lipids identified (Fig. 7A), respectively, including their ether forms, namely PC(O-) and PE(O-). PI, PS, and SM were detected in amounts of 5.61%, 2.68%, and 1.47%, respectively. The lowest content was found for HexCer, LPE, PG, Cer, and LPC classes. The concentrations of individual lipid classes determined in mouse pancreatic tissue samples, with respect to sex, are presented in Fig. 7B. Interestingly, among all analyzed classes, only for HexCer, a statistically significant difference between female and male individuals was observed. A higher content of identified HexCer species was found in the female pancreatic tissue and amounted to 24.67 ± 3.00 pmol/mg wet tissue, whereas in the male pancreatic tissue, it was 6.39 ± 1.64 pmol/mg wet tissue.

Finally, the concentrations of individual lipid species identified in the female and male mouse pancreatic tissues are summarized in Supplemental Table S6. The data reveal, with only minor exceptions, similar quantitative trends between sexes. The most abundant polar lipid species in both female and male samples were PC 34:2 and PC 36:4, each reaching concentrations of approximately 8 nmol/mg of wet tissue. Among PE species, PE O-36:5 and PE 38:4 were the most prevalent, with average concentrations of about 4.0 and 2.8 nmol/mg of wet tissue, respectively. Within the PI class, PI 38:4 showed a markedly higher concentration (2.4 nmol/mg of wet tissue) compared with other PI species. Consistent with the higher total HexCer content observed in female pancreatic tissue, all individual HexCer species, including HexCer 42:1, HexCer 40:1, HexCer 34:1, HexCer 42:2, and HexCer 38:1, were also more abundant in females. Notably, HexCer 38:1 had the lowest concentration in male pancreatic tissue at 0.79 pmol/mg of wet tissue. In contrast, the least abundant species in female tissue were PC 33:0, Cer 36:2;O2, and PI 32:0 with concentrations of 1.2, 1.4, and 1.7 pmol/mg of wet tissue, respectively.

## Discussion

Compared with biofluids, tissue samples appear to be more metabolically active; thus, their processing may require special handling conditions. Our initial experiments conducted at room temperature revealed substantial intersample variations among individual samples of porcine pancreas, which we attribute to the relatively high activity of lipolytic enzymes at this temperature. Therefore, in phase I, the stability of porcine pancreatic tissue stored on ice for up to 120 min was evaluated (Fig. 1). Lipidomic profiling revealed a pronounced, time-dependent formation of lyso-PLs, most likely mediated by PLA_2_ (Fig. 2C). However, it is intriguing that a statistically significant decrease in peak areas of GPLs between control and 120 min of tissue storage on ice was observed for only a few species, among which only PE 36:3 exhibited a relatively high abundance in the analyzed tissue (Supplemental Table S2). This phenomenon may be attributed to the considerably higher levels of precursor GPLs compared with initial amounts of lyso-PLs in the tested samples. For example, the ratio of the total peak area of PCs to LPCs exceeded 100, whereas the ratio of PEs to LPEs was approximately 50 (Supplemental Table S1). Consequently, even a small degree of GPL hydrolysis catalyzed by PLA_2_ may result in a substantial relative increase in lyso-PL levels during sample storage on ice.

In pancreatic tissue, PLA_2_ is initially present as an inactive zymogen and is activated upon proteolytic cleavage (49). Previous studies with porcine pancreatic homogenate showed its increased activity after prolonged room-temperature storage (37). The authors suggested that the increase in enzyme activity might result from proteolytic autolysis toward zymogen. In our study, a progressive increase in lyso-PL formation was observed during 2-h storage of intact, nonhomogenized tissue samples on ice, which suggests that a similar enzyme activation process may also occur at lower temperatures. Previous research has also shown that porcine pancreatic PLA_2_ displayed about four times higher activity toward PE than PC, when both GPL classes contain oleic acid at *sn*-1 and *sn*-2 positions (50). This substrate preference may underlie the statistically significant increase in peak areas for LPE and LPE(O-) observed as early as 60 min of tissue storage on ice, whereas choline-containing lyso-PLs showed significant changes only after 120 min (Fig. 2C). Furthermore, based on the literature data, the hydrolysis rate tends to decrease when saturated fatty acids are incorporated into the GPL structure (50). This may account for the fact that only unsaturated GPLs, namely PC 38:6, PC 40:9, PE 36:3, and PE O-33:3, exhibited a statistically significant decrease in peak areas between the control and 120 min of storage under our experimental conditions. It should be noted that a full understanding of how the fatty acid structure of precursor GPLs influences their hydrolysis kinetics may require identification at the molecular lipid species level, which can be achieved using a different separation mode and/or tandem MS.

Data obtained in phase I indicate that the use of ice is sufficient to preserve lipidome stability, provided that tissue samples are handled within 30 min. However, exceeding this time appears to result in lipid alterations, particularly an artificial increase in lyso-PL levels. This observation prompted us to also investigate the effect of handling temperature on pancreatic tissue stability. For this purpose, porcine and mouse pancreatic tissues were handled either in liquid nitrogen or on ice for comparison. The deep-freezing approach should allow for rapid inhibition of hydrolytic enzyme activity and minimize chemical degradation. From a technical point of view, deep-frozen tissue samples (e.g., snap frozen in liquid nitrogen) can be crushed into small pieces or ground into powder using a pestle and stainless steel mortar cooled with liquid nitrogen (51). Moreover, it can be a good practice to wrap the frozen tissue in aluminum foil before crushing to avoid crosscontamination and sample loss (52).

In our workflow, instead of a mortar and pestle, an aluminum block immersed in liquid nitrogen up to its upper edge was enabled. First, this approach helped maintain consistently low tissue temperature by processing the sample near the liquid/vapor nitrogen interface. Second, the flat surface of the aluminum block facilitated easier tissue handling with tools during sample aliquoting. Tissue aliquoting can be performed directly before homogenization and extraction, or aliquoted and weighed samples can be stored at −80 °C prior to further steps. In both cases, in addition to the aliquoting temperature, attention must also be paid to the temperature during intermediate sample storage (e.g., while the samples from the batch are waiting for aliquoting or for the addition of homogenization solvent). Moreover, the size of the batch being processed affects the duration of the intermediate sample storage, and if the temperature is not sufficiently low, lipidome alterations may occur. In phase II of our experiment, two handling approaches were compared using porcine and mice tissue samples. The approaches differed specifically in the medium used during the handling step, from tissue removal from the −80°C freezer to homogenization, either on ice or in liquid nitrogen.

Taking into account the total peak area of individual lipid classes, significant differences between samples processed on ice or in liquid nitrogen were observed only for low-abundant ones. Importantly, the affected lipid classes differed between the two animal species studied. In porcine tissue, samples processed using liquid nitrogen exhibited significantly lower levels of ether lyso-PLs, specifically LPC(O-) and LPE(O-). In contrast, in mouse tissue handled under the same conditions, a higher amount of CE and lower levels of MGs were detected. These findings may reflect species-specific differences in lipidome compositions themselves, as well as lipolytic enzyme activity between the porcine and mouse pancreas. An example is the markedly higher content of ether lyso-PL species in porcine tissue compared with mouse tissue, which allowed us to identify a significant effect of handling temperature on changes in the levels of these lipids. Based on this, PLA_2_ appears to be a crucial enzyme affecting lipidome instability in porcine pancreatic tissue. Moreover, this experiment indicated that not only processing time (as demonstrated in phase I) but also handling temperature influences the formation of lyso-PLs in porcine tissue. In mouse tissue, pancreatic triacylglycerol lipase and Chol esterase appear to play a primary role in lipidome changes during sample handling. This is consistent with the higher CE content (the substrate in Chol esterase-catalyzed hydrolysis) and lower levels of MGs (products of TG and DG hydrolysis) observed in tissue samples handled using liquid nitrogen compared with those processed on ice. The findings suggest that, when analyzing the same organ/tissue type, but originating from various species, distinct lipid classes can contribute to lipidome instability during sample processing. This suggestion is supported by the presence of various orthologs of individual lipolytic enzymes in the pancreas of various species, which differ in their biochemical properties and enzymatic activity (53).

It should also be noted that the relatively small sample size used in phase II (six samples processed on ice—three porcine and three mouse tissues—and six in liquid nitrogen), which resulted in brief handling times, likely constrained the detectable lipidome changes. In larger batches, prolonged processing can increase degradation risks. Furthermore, ice-based handling increases the risk of partial tissue thawing. If samples are not immediately extracted but returned to −80°C, an additional freeze-thaw cycle may introduce further variability. In our study, samples processed on ice were directly extracted, avoiding this issue.

The individual precursor lipid species that appeared to drive the separation of samples handled on ice versus in liquid nitrogen in PCA (specifically PC 38:1, PC 32:2, PE O-36:5, and TG 52:4 in porcine tissue, as well as TG 54:4, TG 50:2, and TG 47:0 in mouse tissue) most likely result from the heterogeneous distribution of analytes within pancreatic tissue, a common feature of such biological samples (24). This conclusion is based on the absence of a clear trend between the levels of these precursor compounds and the handling temperature, in contrast to the consistent patterns observed in total peak areas of individual lipid classes (Fig. 3), where lower processing temperature was associated with reduced levels of enzymatic degradation products and elevated content of precursor class. Examples of lipids exhibiting spatial variation within pancreatic tissue are neutral lipids, which are predominantly stored in adipocytes that infiltrate the pancreatic parenchyma (40). As a consequence, the tissue is characterized by pronounced heterogeneity, even at the microscopic level, as shown by Rugivarodom *et al*. (54) (Supplemental Fig. S5).

As the qualitative and quantitative characterization of polar lipid classes was of main interest of our study, in phase III, the fractionation step of total pancreatic tissue lipid extracts was implemented in our sample processing workflow. LLE using hexane and MeOH was employed in order to fractionate lipid extracts. To optimize partitioning efficiency between polar and nonpolar lipid classes, the water content in MeOH was adjusted. Based on experimental data, 2% (v/v) water in MeOH was selected for further work, as it effectively reduced CE and TG in the MeOH-rich phase. Nevertheless, neutral lipid removal efficiency also depended on their initial abundance, and in extracts with high nonpolar lipid content, larger solvent volumes or multiple extraction cycles may be required.

Solvent composition plays a crucial role in lipid partitioning. For example, in a study focused on human adipose tissue lipidome characterization, the fractionation was performed by a multiple LLE step with an ethanol/water mixture (87:13 v/v) and hexane (25). However, due to the lower polarity of ethanol and high TG content in adipose tissue, this lipid class was not fully removed from the polar fraction. In our case, Cer species and Chol were particularly sensitive to water content in MeOH, showing reduced affinity for the polar phase as the water content in MeOH increased. A change from 0% to 2% (v/v) water resulted in a 25.7% reduction in total Cer signal, whereas 5% water caused a 57.1% decrease compared with anhydrous MeOH. Despite this loss, the reduced presence of TG enabled the use of lower dilution factors, improving Cer detectability. Moreover, no systematic effect of Cer fatty acyl chain length or saturation on phase distribution was observed. For other polar lipid classes, water addition had minimal or slightly positive effects on partitioning behavior.

The developed protocol was finally applied to study pancreatic tissue polar lipidomes of female and male mice. In total, we identified 209 polar lipid species in both sexes (Supplemental Table S5). In comparison, 256 lipid species were identified in the mouse pancreas in a previous study investigating the effect of chronic jetlag on the organ lipidome (55). However, this number also included nonpolar lipids, such as TG and DG, as well as other classes not included in our study, such as fatty acids or fatty acid esters of hydroxy fatty acids. In another study evaluating different extraction protocols in six murine organs, 709 lipid species were detected in pooled mouse pancreatic samples, including 233 neutral lipids (56). However, the identification was performed on a pooled sample of pancreatic tissue extracts obtained using all tested extraction methods. Considering the use of solvents with different polarities and protocols involving single-, two-, or three-phase extraction methods, a wide range of lipid classes and species was covered. In addition, the lipids in the referenced study were separated using the reverse-phase LC, where retention depends on both fatty acyl chain length and the number and position of double bonds (17). This allows for higher separation efficiency, and consequently, the identification of a greater number of lipid species. However, reverse-phase mode typically requires a longer analysis time; for example, the referenced study reported a 32-min run time (56), compared with 8 min in our approach.

Absolute quantitative data on tissue lipidomes remain limited due to analytical complexity and cost. Most studies report relative values, which complicates interlaboratory comparisons. The rodent liver is the organ most frequently studied, providing more reference data. For example, the concentrations of total PC and PE in the liver of normal-chow mice were found to be around 15 and 30 nmol/mg of tissue, respectively (57), whereas in the rat liver, it was 31.2 and 22.8 nmol/mg of tissue, respectively (58). In the case of our study, the concentrations of the five most abundant polar lipid classes, namely PC, PE, PI, PS, and SM, were as follows: 31.1, 15.5, 3.0, 1.4, and 0.8 nmol/mg of tissue. Importantly, the ratio between the two most abundant PL classes (PC and PE) in our study is closely correlated with that reported in the study that evaluated different extraction protocols in six mouse organs, including the pancreas (56). However, in this study, the authors expressed the amounts as molar concentration, which makes it difficult to directly compare the data obtained. Furthermore, the lipid composition presented in the study did not indicate the presence of PI and PS classes, which, according to our data, are the most abundant polar lipid classes after PC and PE. The referenced study also showed high levels of LPC and LPE, representing approximately 10% and 20% of their precursor PL, respectively (56). In our study, these ratios were 1.9% and 0.6%, respectively, suggesting a lower degree of PC and PE degradation during the workflow. This difference may be attributed to tissue homogenization in an aqueous solution, which could allow PLA_2_ to remain enzymatically active. However, it should be noted that in both cases, the homogenization was carried out at a reduced temperature (<5°C) using the Cryolys® Evolution cooling unit.

The quantitative analysis of polar lipid classes in pancreatic tissues did not reveal significant differences between sexes, except for the HexCer class, which was nearly four times more abundant in females compared with males. To date, sex-specific differences in sphingolipid classes have been documented in different organs. For example, Muralidharan *et al*. (59) found the greatest differences in kidneys, mainly due to Hex2Cer levels, and observed higher SM concentrations in female livers. However, the cited study (59), despite taking into account 21 different tissue types, did not include the pancreas. Furthermore, to the best of our knowledge, other studies also did not report sex-specific differences in the pancreatic lipidome.

In summary, we have developed a reliable preanalytical workflow for the processing of pancreatic tissues and subsequent MS-based lipidomic analysis, enabling the identification of 209 polar lipid species across 10 lipid classes in the mouse pancreas. Rapid snap-freezing upon collection and continuous maintenance of ultra-low temperatures during subsequent handling are critical to prevent lipid degradation due to the high metabolic activity of pancreatic tissues. Our study showed that storing porcine pancreatic tissue on ice for 60–120 min led to significant formation of lyso-PLs likely driven by PLA_2_ activity. To avoid such artifacts, we recommend the use of liquid nitrogen during sample aliquoting and intermediate storage. Furthermore, the high content of nonpolar lipids, such as TGs, can lead to chromatographic overloading, carryover, and contamination of the mass spectrometer. The application of lipid class fractionation, aimed at the removal of nonpolar lipid, enabled the injection of higher concentrations of polar lipid fractions. Consequently, in phase IV of the study, it was possible to detect and quantify five HexCer species in mouse pancreatic tissue that were not detected when total lipid extracts were analyzed in phase II. This optimized preanalytical workflow provides a reproducible approach for tissue lipidomics, supporting lipid biomarker discovery and advancing our understanding of lipid metabolism in biomedical research.

## Data availability

All data are contained in the article and the accompanying supplemental data.

## Supplemental data

This article contains supplemental data (54).

## Conflict of interests

The authors declare that they have no conflicts of interest with the contents of this article.
