# Supplementary material for: Improved preanalytical workflow for pancreatic tissue lipidomics: insights into lipid stability and polar lipid recovery
Source: J Lipid Res. 2025 Dec 26;67(2):100968. doi: 10.1016/j.jlr.2025.100968 (PMC12828829; doi:10.1016/j.jlr.2025.100968)
Supplement: Supplemental Data 1 [file mmc1.pdf]

Contents of Report

Created by https://lipidomicstandards.org, version v2.5.0

Separation Workflow 1
Overall study design 1
Lipid extraction 1
Analytical platform 2
Quality control 2
Method qualification and validation 2
Reporting 2

Sample Descriptions 2
Pancreas_Porcine / Pig / Tissues (e.g., liver, heart, brain) 2
Pancreas_Mouse / Mouse / Tissues (e.g., liver, heart, brain) 3

Lipid Class Descriptions 3
1) Cer[M+H-H2O]+ / Lipid identification 3
1) Cer[M+H-H2O]+ / Lipid quantification 3
2) HexCer[M+CH3COO]- / Lipid identification 4
2) HexCer[M+CH3COO]- / Lipid quantification 4
3) LPC[M+CH3COO]- / Lipid identification 4
3) LPC[M+CH3COO]- / Lipid quantification 5
4) LPE[M-H]- / Lipid identification 5
4) LPE[M-H]- / Lipid quantification 5
5) LPE O[M-H]- / Lipid identification 6
5) LPE O[M-H]- / Lipid quantification 6
6) PC[M+H]+ / Lipid identification 6
6) PC[M+H]+ / Lipid quantification 7
7) PC O[M+H]+ / Lipid identification 7
7) PC O[M+H]+ / Lipid quantification 8
8) PE[M-H]- / Lipid identification 8
8) PE[M-H]- / Lipid quantification 8
9) PE O[M+H]+ / Lipid identification 9
9) PE O[M+H]+ / Lipid quantification 9
10) PG[M-H]- / Lipid identification 10
10) PG[M-H]- / Lipid quantification 10
11) PI[M-H]- / Lipid identification 10
11) PI[M-H]- / Lipid quantification 11
12) PS[M-H]- / Lipid identification 11
12) PS[M-H]- / Lipid quantification 11
13) SM[M+H]+ / Lipid identification 12
13) SM[M+H]+ / Lipid quantification 12

Separation Workflow

Overall study design

Title of the study	Improved preanalytical workflow for pancreatic tissue lipidomics: Insights into lipid stability and polar lipid recovery		
Document creation date	10/14/2025	Principal investigator	Michal Holčapek
Institution	Department of Analytical Chemistry, Faculty of Chemical Technology, University of Pardubice, Pardubice, Czech Republic	Corresponding Email	michal.holcapek@upce.cz
Is the workflow targeted or untargeted?	Untargeted	Clinical	No

Lipid extraction

Extraction method	2-phase system	pH adjustment	None
2-phase system	Folch	Special conditions	Sonication in a water bath and intensive mixing, both at room temperature

Were internal standards used?	Yes	Deposition method	The internal standard mixture was added to the chloroform/methanol extraction system prior to the addition of the tissue homogenate
Internal standards used	CE 16:0 d7; Cer C18 d7; Chol d7; DG 33:1 d7; GlcCer 36:1 d5; LPC 18:1 d7; LPE 18:1 d7; MG 18:1 d7; PC 33:1 d7; PC P-36:1 d9; PE 33:1 d7; PE P-36:1 d9; PG 33:1 d7; PI 33:1 d7; PS 33:1 d7; SM 36:2 d9; TG 48:1 d7; CE 17:0; Cer 35:1; DG 36:2 d5; GlcCer 30:1; LPC 13:0; LPE 14:0; MG 19:1; PC 28:0; PE 28:0; PG 28:0; PI 33:1; PS 28:0; SM 30:1; TG 57:3		

Analytical platform

Ionizaton additives	Ammonium acetate	Number of separation dimensions	One dimension
Separation type 1	SFC	Separation mode 1 (liquid)	NP
Detector	Mass spectrometer	MS type	QTOF
MS vendor	Waters	Ion source	ESI
MS Level	MS^1	Mass resolution for detected ion at MS^1	High resolution
Resolution at m/z 200 at MS^1	40000	Mass accuracy in ppm at MS^1	10
Recording mode of raw data at MS^1	Profile mode	Was/Were additional dimension/techniques used	No

Quality control

Blanks	Yes	Type of Blanks	Extraction blank, Solvent blank
Quality control	Yes	Type of QC sample	Sample pool

Method qualification and validation

Method validation	No

Reporting

Are reported raw data uploaded into repository?	Available on request	Are metadata available?	Available on request
Raw data upload	Available on request		

Sample Descriptions

Pancreas_Porcine / Pig / Tissues (e.g., liver, heart, brain)

Tissue type	Pancreas	Storage and collection conditions	Available
Sample homogenization	No	Provided preanalytical information	Time to freeze, Storage time (month), Freeze-thaw cycles

Temperature handling original sample	N2	Instant sample preparation	No
Time to freeze	between 5 and 120 min	Snap freezing in liquid N2	No
Storage temperature	-80 °C	Storage time (month)	6
Freeze-thaw cycles	1	Additives	None

Pancreas_Mouse / Mouse / Tissues (e.g., liver, heart, brain)

Tissue type	Pancreas	Storage and collection conditions	Available
Sample homogenization	No	Provided preanalytical information	Time to freeze, Storage time (month), Freeze-thaw cycles
Temperature handling original sample	N2	Instant sample preparation	No
Time to freeze	between 5 and 30 min	Snap freezing in liquid N2	Yes
Storage temperature	-80 °C	Storage time (month)	6
Freeze-thaw cycles	0	Additives	None

Lipid Class Descriptions

1) Cer[M+H-H2O]+ / Lipid identification

Lipid class	Cer	MS Level for identification	MS^1
Identification level	Species level	MS^1 adduct	[M+H-H2O]+
Isotope correction at MS^1	Type 2	MS^1 verified by standard	Yes
Background check at MS^1	Yes	Did you presume assumptions for identification?	No
Check on:	Isomeric overlap, Isobaric overlap, In-source fragmentation	Limit of detection	Signal theshold
RT verified by standard	Yes	Separation of isobaric/isomeric interferece confirmed	No
Model for separation prediction	No	Lipid Identification Software	Homemade
Data manipulation	Centroiding, Lock mass correction	Nomenclature for intact lipid molecule	Yes

1) Cer[M+H-H2O]+ / Lipid quantification

Quantitative	Yes	MS Level for quantification	MS^1

Internal lipid standard(s) MS^1

Internal standard	Endogenous subclass
Cer C18 d7	Cer 34:2;O2
Cer C18 d7	Cer 34:1;O2
Cer C18 d7	Cer 36:2;O2
Cer C18 d7	Cer 38:2;O2
Cer C18 d7	Cer 38:1;O2
Cer C18 d7	Cer 40:2;O2
Cer C18 d7	Cer 40:1;O2
Cer C18 d7	Cer 41:2;O2
Cer C18 d7	Cer 41:1;O2
Cer C18 d7	Cer 42:2;O2
Cer C18 d7	Cer 42:1;O2
Cer C18 d7	Cer 40:1;O3
Cer C18 d7	Cer 42:2;O3

Cer C18 d7	Cer 42:1;O3

Type of quantification	Internal standard amount	Response correction	No
Type I isotope correction	Yes	Limit of quantification	Signal theshold
Normalization to reference	No	Lipid Quantification Software	Homemade
Batch correction	No		

2) HexCer[M+CH3COO]- / Lipid identification

Lipid class	HexCer	MS Level for identification	MS^1
Identification level	Species level	MS^1 adduct	[M+CH3COO]-
Isotope correction at MS^1	Type 2	MS^1 verified by standard	Yes
Background check at MS^1	Yes	Did you presume assumptions for identification?	No
Check on:	Isomeric overlap, Isobaric overlap, In-source fragmentation	Limit of detection	Signal theshold
RT verified by standard	Yes	Separation of isobaric/isomeric interferece confirmed	No
Model for separation prediction	No	Lipid Identification Software	Homemade
Data manipulation	Centroiding, Lock mass correction	Nomenclature for intact lipid molecule	Yes

2) HexCer[M+CH3COO]- / Lipid quantification

Quantitative	Yes	MS Level for quantification	MS^1

Internal lipid standard(s) MS^1

Internal standard	Endogenous subclass
GlcCer 36:1 d5	HexCer 34:1
GlcCer 36:1 d5	HexCer 38:1
GlcCer 36:1 d5	HexCer 40:1
GlcCer 36:1 d5	HexCer 42:2
GlcCer 36:1 d5	HexCer 42:1

Type of quantification	Internal standard amount	Response correction	No
Type I isotope correction	Yes	Limit of quantification	Signal theshold
Normalization to reference	No	Lipid Quantification Software	Homemade
Batch correction	No		

3) LPC[M+CH3COO]- / Lipid identification

Lipid class	LPC	MS Level for identification	MS^1
Identification level	Species level	MS^1 adduct	[M+CH3COO]-
Isotope correction at MS^1	Type 2	MS^1 verified by standard	Yes
Background check at MS^1	Yes	Did you presume assumptions for identification?	No
Check on:	Isomeric overlap, Isobaric overlap, In-source fragmentation	Limit of detection	Signal theshold

RT verified by standard	Yes	Separation of isobaric/isomeric interferece confirmed	No
Model for separation prediction	No	Lipid Identification Software	Homemade
Data manipulation	Centroiding, Lock mass correction	Nomenclature for intact lipid molecule	Yes

3) LPC[M+CH3COO]- / Lipid quantification

Quantitative	Yes	MS Level for quantification	MS^1

Internal lipid standard(s) MS^1

Internal standard	Endogenous subclass
LPC 18:1 d7	LPC 15:0
LPC 18:1 d7	LPC 16:0
LPC 18:1 d7	LPC 17:1
LPC 18:1 d7	LPC 17:0
LPC 18:1 d7	LPC 18:2
LPC 18:1 d7	LPC 18:1
LPC 18:1 d7	LPC 18:0
LPC 18:1 d7	LPC 20:4

Type of quantification	Internal standard amount	Response correction	No
Type I isotope correction	Yes	Limit of quantification	Signal theshold
Normalization to reference	No	Lipid Quantification Software	Homemade
Batch correction	No		

4) LPE[M-H]- / Lipid identification

Lipid class	LPE	MS Level for identification	MS^1
Identification level	Species level	MS^1 adduct	[M-H]-
Isotope correction at MS^1	Type 2	MS^1 verified by standard	Yes
Background check at MS^1	Yes	Did you presume assumptions for identification?	No
Check on:	Isomeric overlap, Isobaric overlap, In-source fragmentation	Limit of detection	Signal theshold
RT verified by standard	Yes	Separation of isobaric/isomeric interferece confirmed	No
Model for separation prediction	No	Lipid Identification Software	Homemade
Data manipulation	Centroiding, Lock mass correction	Nomenclature for intact lipid molecule	Yes

4) LPE[M-H]- / Lipid quantification

Quantitative	Yes	MS Level for quantification	MS^1

Internal lipid standard(s) MS^1

Internal standard	Endogenous subclass
LPE 18:1 d7	LPE 16:0
LPE 18:1 d7	LPE 18:1
LPE 18:1 d7	LPE 18:0
LPE 18:1 d7	LPE 20:4

Internal standard	Endogenous subclass
LPE 18:1 d7	LPE 22:6

Type of quantification	Internal standard amount	Response correction	No
Type I isotope correction	Yes	Limit of quantification	Signal theshold
Normalization to reference	No	Lipid Quantification Software	Homemade
Batch correction	No		

5) LPE O[M-H]- / Lipid identification

Lipid class	LPE O	MS Level for identification	MS^1
Identification level	Species level	MS^1 adduct	[M-H]-
Isotope correction at MS^1	Type 2	MS^1 verified by standard	Yes
Background check at MS^1	Yes	Did you presume assumptions for identification?	No
Check on:	Isomeric overlap, Isobaric overlap, In-source fragmentation	Limit of detection	Signal threshold
RT verified by standard	Yes	Separation of isobaric/isomeric interferece confirmed	No
Model for separation prediction	No	Lipid Identification Software	Homemade
Data manipulation	Centroiding, Lock mass correction	Nomenclature for intact lipid molecule	Yes

5) LPE O[M-H]- / Lipid quantification

Quantitative	Yes	MS Level for quantification	MS^1

Internal lipid standard(s) MS^1

Internal standard	Endogenous subclass
LPE 18:1 d7	LPE O-16:1

Type of quantification	Internal standard amount	Response correction	No
Type I isotope correction	Yes	Limit of quantification	Signal threshold
Normalization to reference	No	Lipid Quantification Software	Homemade
Batch correction	No		

6) PC[M+H]+ / Lipid identification

Lipid class	PC	MS Level for identification	MS^1
Identification level	Species level	MS^1 adduct	[M+H]+
Isotope correction at MS^1	Type 2	MS^1 verified by standard	Yes
Background check at MS^1	Yes	Did you presume assumptions for identification?	No
Check on:	Isomeric overlap, Isobaric overlap, In-source fragmentation	Limit of detection	Signal threshold
RT verified by standard	Yes	Separation of isobaric/isomeric interferece confirmed	No
Model for separation prediction	No	Lipid Identification Software	Homemade

Data manipulation	Centroiding, Lock mass correction	Nomenclature for intact lipid molecule	Yes

6) PC[M+H]+ / Lipid quantification

Quantitative	Yes	MS Level for quantification	MS^1

Internal lipid standard(s) MS^1

Internal standard	Endogenous subclass
PC 33:1 d7	PC 31:0
PC 33:1 d7	PC 32:2
PC 33:1 d7	PC 32:0
PC 33:1 d7	PC 33:2
PC 33:1 d7	PC 33:1
PC 33:1 d7	PC 33:0
PC 33:1 d7	PC 34:3
PC 33:1 d7	PC 34:2
PC 33:1 d7	PC 34:1
PC 33:1 d7	PC 36:4
PC 33:1 d7	PC 36:3
PC 33:1 d7	PC 36:2
PC 33:1 d7	PC 38:6
PC 33:1 d7	PC 38:5
PC 33:1 d7	PC 38:4
PC 33:1 d7	PC 38:3
PC 33:1 d7	PC 39:4
PC 33:1 d7	PC 40:8
PC 33:1 d7	PC 40:7
PC 33:1 d7	PC 40:5

Type of quantification	Internal standard amount	Response correction	No
Type I isotope correction	Yes	Limit of quantification	Signal threshold
Normalization to reference	No	Lipid Quantification Software	Homemade
Batch correction	No		

7) PC O[M+H]+ / Lipid identification

Lipid class	PC O	MS Level for identification	MS^1
Identification level	Species level	MS^1 adduct	[M+H]+
Isotope correction at MS^1	Type 2	MS^1 verified by standard	Yes
Background check at MS^1	Yes	Did you presume assumptions for identification?	No
Check on:	Isomeric overlap, Isobaric overlap, In-source fragmentation	Limit of detection	Signal threshold
RT verified by standard	Yes	Separation of isobaric/isomeric interferece confirmed	No
Model for separation prediction	No	Lipid Identification Software	Homemade
Data manipulation	Centroiding, Lock mass correction	Nomenclature for intact lipid molecule	Yes

7) PC O[M+H]+ / Lipid quantification

Quantitative	Yes	MS Level for quantification	MS^1

Internal lipid standard(s) MS^1

Internal standard	Endogenous subclass
PC P-36:1 d9	PC O-34:3
PC P-36:1 d9	PC O-38:7
PC P-36:1 d9	PC O-38:6

Type of quantification	Internal standard amount	Response correction	No
Type I isotope correction	Yes	Limit of quantification	Signal threshold
Normalization to reference	No	Lipid Quantification Software	Homemade
Batch correction	No		

8) PE[M-H]- / Lipid identification

Lipid class	PE	MS Level for identification	MS^1
Identification level	Species level	MS^1 adduct	[M-H]-
Isotope correction at MS^1	Type 2	MS^1 verified by standard	Yes
Background check at MS^1	Yes	Did you presume assumptions for identification?	No
Check on:	Isomeric overlap, Isobaric overlap, In-source fragmentation	Limit of detection	Signal threshold
RT verified by standard	Yes	Separation of isobaric/isomeric interferece confirmed	No
Model for separation prediction	No	Lipid Identification Software	Homemade
Data manipulation	Centroiding, Lock mass correction	Nomenclature for intact lipid molecule	Yes

8) PE[M-H]- / Lipid quantification

Quantitative	Yes	MS Level for quantification	MS^1

Internal lipid standard(s) MS^1

Internal standard	Endogenous subclass
PE 33:1 d7	PE 32:1
PE 33:1 d7	PE 32:0
PE 33:1 d7	PE 34:3
PE 33:1 d7	PE 34:2
PE 33:1 d7	PE 34:1
PE 33:1 d7	PE 36:5
PE 33:1 d7	PE 36:4
PE 33:1 d7	PE 36:3
PE 33:1 d7	PE 36:2
PE 33:1 d7	PE 36:1
PE 33:1 d7	PE 38:6
PE 33:1 d7	PE 38:5
PE 33:1 d7	PE 38:4
PE 33:1 d7	PE 38:3
PE 33:1 d7	PE 39:4
PE 33:1 d7	PE 40:7

PE 33:1 d7	PE 40:6
PE 33:1 d7	PE 40:5
PE 33:1 d7	PE 40:4
PE 33:1 d7	PE 42:6

Type of quantification	Internal standard amount	Response correction	No
Type I isotope correction	Yes	Limit of quantification	Signal threshold
Normalization to reference	No	Lipid Quantification Software	Homemade
Batch correction	No		

9) PE O[M+H]+ / Lipid identification

Lipid class	PE O	MS Level for identification	MS^1
Identification level	Species level	MS^1 adduct	[M+H]+
Isotope correction at MS^1	Type 2	MS^1 verified by standard	Yes
Background check at MS^1	Yes	Did you presume assumptions for identification?	No
Check on:	Isomeric overlap, Isobaric overlap, In-source fragmentation	Limit of detection	Signal threshold
RT verified by standard	Yes	Separation of isobaric/isomeric interferece confirmed	No
Model for separation prediction	No	Lipid Identification Software	Homemade
Data manipulation	Centroiding, Lock mass correction	Nomenclature for intact lipid molecule	Yes

9) PE O[M+H]+ / Lipid quantification

Quantitative	Yes	MS Level for quantification	MS^1

Internal lipid standard(s) MS^1

Internal standard	Endogenous subclass
PE P-36:1 d9	PE O-34:2
PE P-36:1 d9	PE O-36:5
PE P-36:1 d9	PE O-36:3
PE P-36:1 d9	PE O-38:7
PE P-36:1 d9	PE O-38:5
PE P-36:1 d9	PE O-38:4
PE P-36:1 d9	PE O-39:3
PE P-36:1 d9	PE O-40:9
PE P-36:1 d9	PE O-40:8
PE P-36:1 d9	PE O-40:7
PE P-36:1 d9	PE O-40:6

Type of quantification	Internal standard amount	Response correction	No
Type I isotope correction	Yes	Limit of quantification	Signal threshold
Normalization to reference	No	Lipid Quantification Software	Homemade
Batch correction	No		

10) PG[M-H]- / Lipid identification

Lipid class	PG	MS Level for identification	MS^1
Identification level	Species level	MS^1 adduct	[M-H]-
Isotope correction at MS^1	Type 2	MS^1 verified by standard	Yes
Background check at MS^1	Yes	Did you presume assumptions for identification?	No
Check on:	Isomeric overlap, Isobaric overlap, In-source fragmentation	Limit of detection	Signal threshold
RT verified by standard	Yes	Separation of isobaric/isomeric interferece confirmed	No
Model for separation prediction	No	Lipid Identification Software	Homemade
Data manipulation	Centroiding, Lock mass correction	Nomenclature for intact lipid molecule	Yes

10) PG[M-H]- / Lipid quantification

Quantitative	Yes	MS Level for quantification	MS^1

Internal lipid standard(s) MS^1

Internal standard	Endogenous subclass
PG 33:1 d7	PG 34:2
PG 33:1 d7	PG 34:1
PG 33:1 d7	PG 36:2
PG 33:1 d7	PG 36:1
PG 33:1 d7	PG 38:4
PG 33:1 d7	PG 40:8
PG 33:1 d7	PG 40:7
PG 33:1 d7	PG 40:6
PG 33:1 d7	PG 42:9

Type of quantification	Internal standard amount	Response correction	No
Type I isotope correction	Yes	Limit of quantification	Signal threshold
Normalization to reference	No	Lipid Quantification Software	Homemade
Batch correction	No		

11) PI[M-H]- / Lipid identification

Lipid class	PI	MS Level for identification	MS^1
Identification level	Species level	MS^1 adduct	[M-H]-
Isotope correction at MS^1	Type 2	MS^1 verified by standard	Yes
Background check at MS^1	Yes	Did you presume assumptions for identification?	No
Check on:	Isomeric overlap, Isobaric overlap, In-source fragmentation	Limit of detection	Signal threshold
RT verified by standard	Yes	Separation of isobaric/isomeric interferece confirmed	No
Model for separation prediction	No	Lipid Identification Software	Homemade
Data manipulation	Centroiding, Lock mass correction	Nomenclature for intact lipid molecule	Yes

11) PI[M-H]- / Lipid quantification

Quantitative	Yes	MS Level for quantification	MS^1

Internal lipid standard(s) MS^1

Internal standard	Endogenous subclass
PI 33:1 d7	PI 32:0
PI 33:1 d7	PI 34:2
PI 33:1 d7	PI 34:1
PI 33:1 d7	PI 36:4
PI 33:1 d7	PI 36:3
PI 33:1 d7	PI 36:2
PI 33:1 d7	PI 36:1
PI 33:1 d7	PI 36:0
PI 33:1 d7	PI 37:4
PI 33:1 d7	PI 38:6
PI 33:1 d7	PI 38:5
PI 33:1 d7	PI 38:4
PI 33:1 d7	PI 38:3
PI 33:1 d7	PI 39:4
PI 33:1 d7	PI 40:7
PI 33:1 d7	PI 40:6
PI 33:1 d7	PI 40:5
PI 33:1 d7	PI 40:4

Type of quantification	Internal standard amount	Response correction	No
Type I isotope correction	Yes	Limit of quantification	Signal threshold
Normalization to reference	No	Lipid Quantification Software	Homemade
Batch correction	No		

12) PS[M-H]- / Lipid identification

Lipid class	PS	MS Level for identification	MS^1
Identification level	Species level	MS^1 adduct	[M-H]-
Isotope correction at MS^1	Type 2	MS^1 verified by standard	Yes
Background check at MS^1	Yes	Did you presume assumptions for identification?	No
Check on:	Isomeric overlap, Isobaric overlap, In-source fragmentation	Limit of detection	Signal threshold
RT verified by standard	Yes	Separation of isobaric/isomeric interferece confirmed	No
Model for separation prediction	No	Lipid Identification Software	Homemade
Data manipulation	Centroiding, Lock mass correction	Nomenclature for intact lipid molecule	Yes

12) PS[M-H]- / Lipid quantification

Quantitative	Yes	MS Level for quantification	MS^1

Internal lipid standard(s) MS^1

Internal standard	Endogenous subclass

PS 33:1 d7	PS 36:2
PS 33:1 d7	PS 36:1
PS 33:1 d7	PS 38:4
PS 33:1 d7	PS 40:6
PS 33:1 d7	PS 40:5
PS 33:1 d7	PS 40:4

Type of quantification	Internal standard amount	Response correction	No
Type I isotope correction	Yes	Limit of quantification	Signal threshold
Normalization to reference	No	Lipid Quantification Software	Homemade
Batch correction	No		

13) SM[M+H]+ / Lipid identification

Lipid class	SM	MS Level for identification	MS^1
Identification level	Species level	MS^1 adduct	[M+H]+
Isotope correction at MS^1	Type 2	MS^1 verified by standard	Yes
Background check at MS^1	Yes	Did you presume assumptions for identification?	No
Check on:	Isomeric overlap, Isobaric overlap, In-source fragmentation	Limit of detection	Signal threshold
RT verified by standard	Yes	Separation of isobaric/isomeric interferece confirmed	No
Model for separation prediction	No	Lipid Identification Software	Homemade
Data manipulation	Centroiding, Lock mass correction	Nomenclature for intact lipid molecule	Yes

13) SM[M+H]+ / Lipid quantification

Quantitative	Yes	MS Level for quantification	MS^1

Internal lipid standard(s) MS^1

Internal standard	Endogenous subclass
SM 36:2 d9	SM 34:2
SM 36:2 d9	SM 36:2
SM 36:2 d9	SM 36:1
SM 36:2 d9	SM 41:1
SM 36:2 d9	SM 42:3
SM 36:2 d9	SM 42:2

Type of quantification	Internal standard amount	Response correction	No
Type I isotope correction	Yes	Limit of quantification	Signal threshold
Normalization to reference	No	Lipid Quantification Software	Homemade
Batch correction	No		
