# Supplementary material for: Improved preanalytical workflow for pancreatic tissue lipidomics: insights into lipid stability and polar lipid recovery
Source: J Lipid Res. 2025 Dec 26;67(2):100968. doi: 10.1016/j.jlr.2025.100968 (PMC12828829; doi:10.1016/j.jlr.2025.100968)

Supplementary Data 3

Improved preanalytical workflow for pancreatic tissue lipidomics: Insights into lipid stability and polar lipid recovery

Karol Parchem[1], Malena Manzi[1], Robert Jirásko[1], Ondřej Peterka[1], Zuzana Lásko[1], Ondřej Kuda[2], Michal Holčapek[1,*]

[1]Department of Analytical Chemistry, Faculty of Chemical Technology, University of Pardubice, Pardubice, Czech Republic

[2]Institute of Physiology, Czech Academy of Sciences, Prague, Czech Republic

*Corresponding author: Michal Holčapek, Department of Analytical Chemistry, Faculty of Chemical Technology, University of Pardubice, Studentská 573, 53210 Pardubice, Czech Republic, Michal.Holcapek@upce.cz

Figure S1. Concentrations of individual lysophosphatidylcholine (LPC) and lysophosphatidylethanolamine (LPE) species in sequential samples collected from two distinct regions of the porcine pancreas (region 1 – samples 1–3; region 2 – samples 4–6).

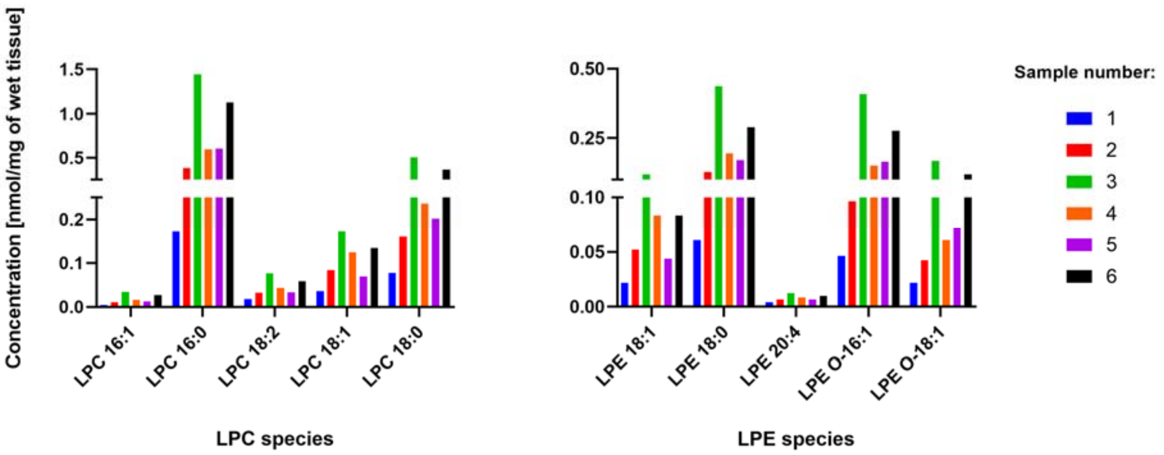

Figure S2. Two-dimensional (2D) PCA score plots of total lipid extracts from porcine (A, B) and mouse (C, D) pancreatic tissue samples processed using either ice or liquid nitrogen. Plots show different principal component combinations: PC1-PC2 (A, C) and PC1-PC3 (B, D). Biplots presenting scores and loadings for total lipid extracts from porcine (E) and mouse (F) pancreatic tissue, based on PC2 and PC3.

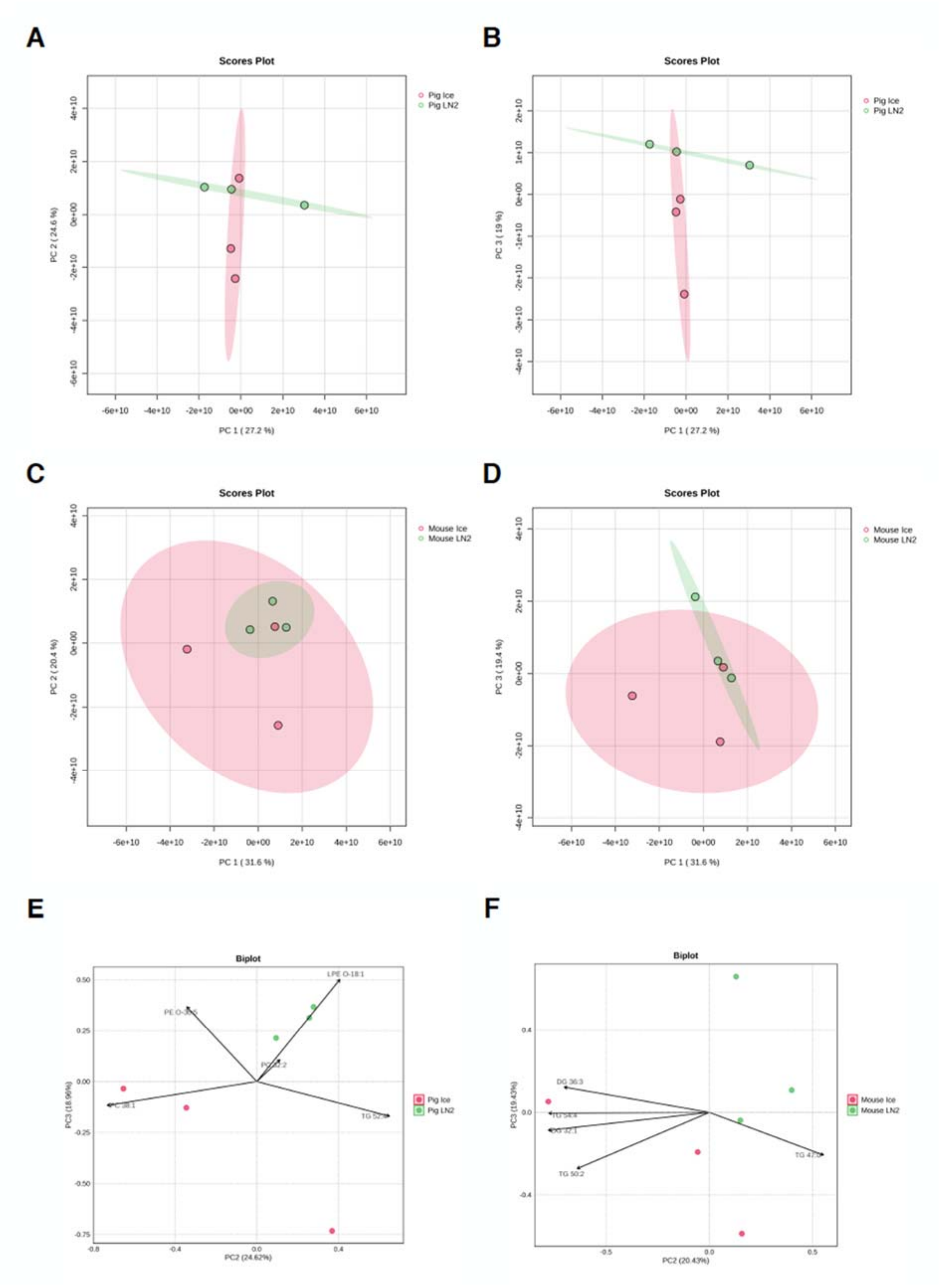

Figure S3. Total peak areas of individual deuterated internal standards detected in the MeOH- and Hex-rich phases depending on the water content in MeOH used during the fractionation of total pancreatic tissue lipid extracts. The values are mean of three independent measurements.

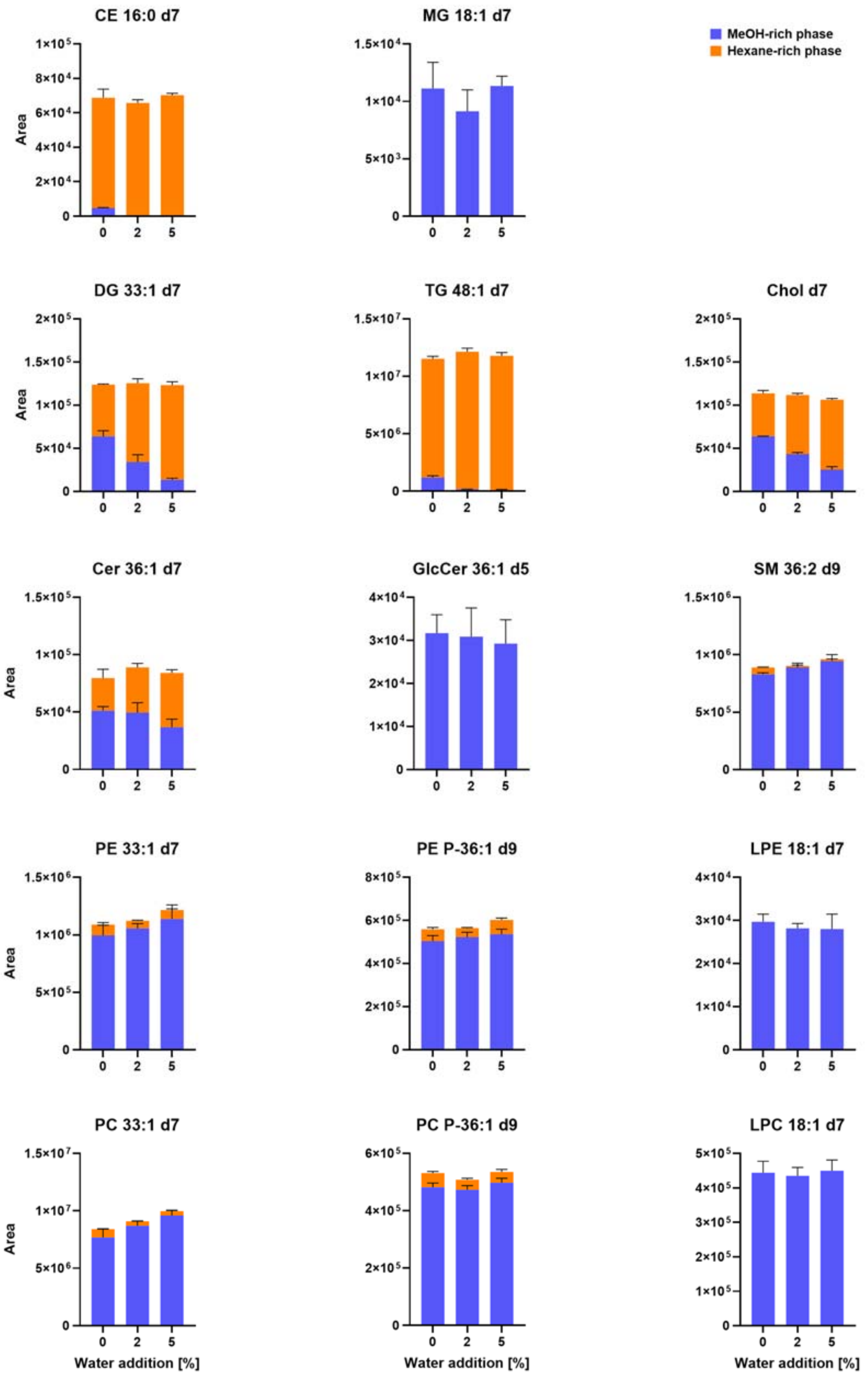

Figure S4. Total peak areas of individual Cer species detected in the MeOH- and Hex-rich phases depending on the water content in MeOH used during the fractionation of total pancreatic tissue lipid extracts. The values are mean of three independent measurements.

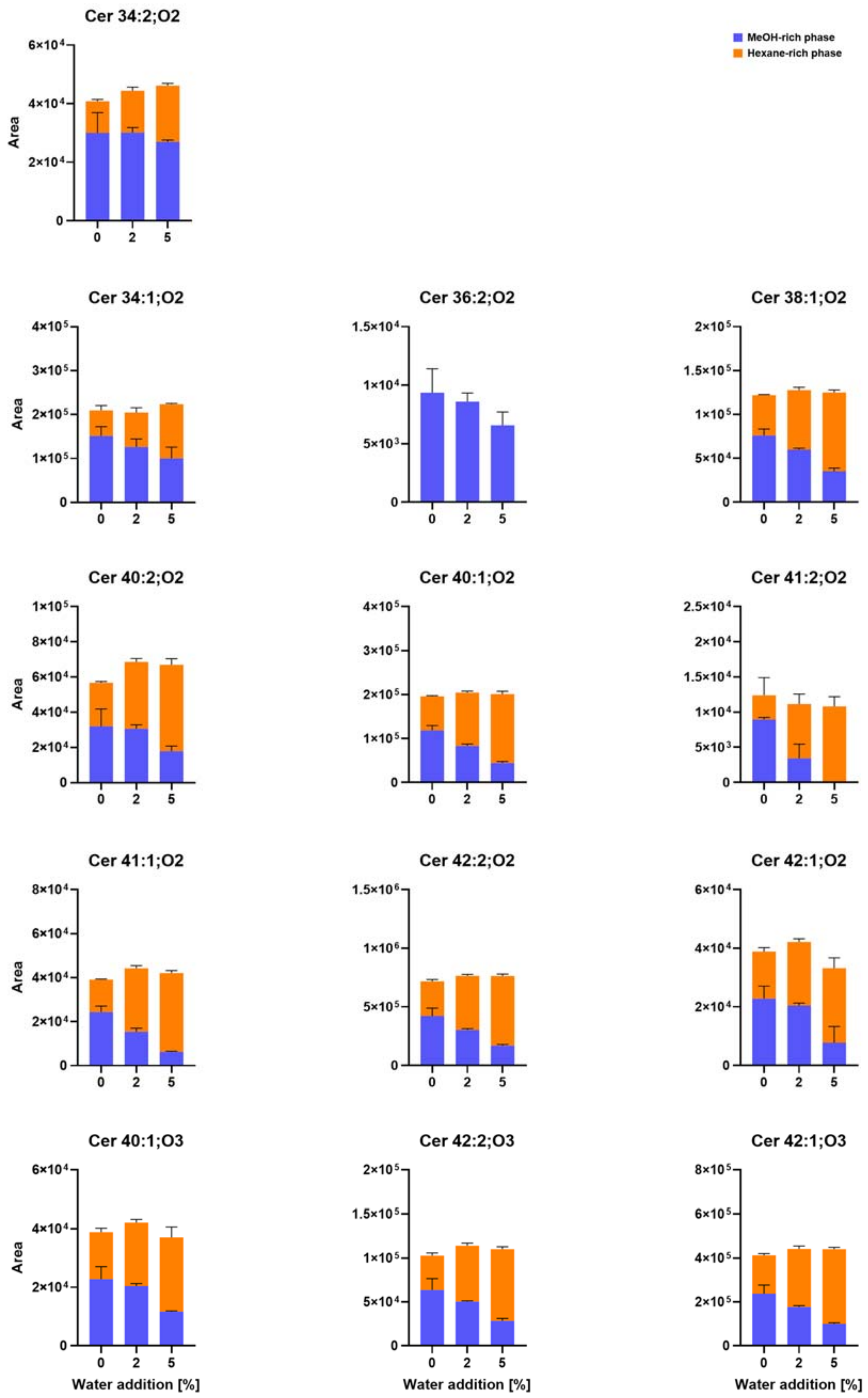

Figure S5. Total peak areas of PG, PI, and PS species and deuterated internal standards corresponding to these lipid classes detected in the MeOH-rich phases depending on the water content in MeOH used during the fractionation of total pancreatic tissue lipid extracts. The values are mean of three independent measurements.

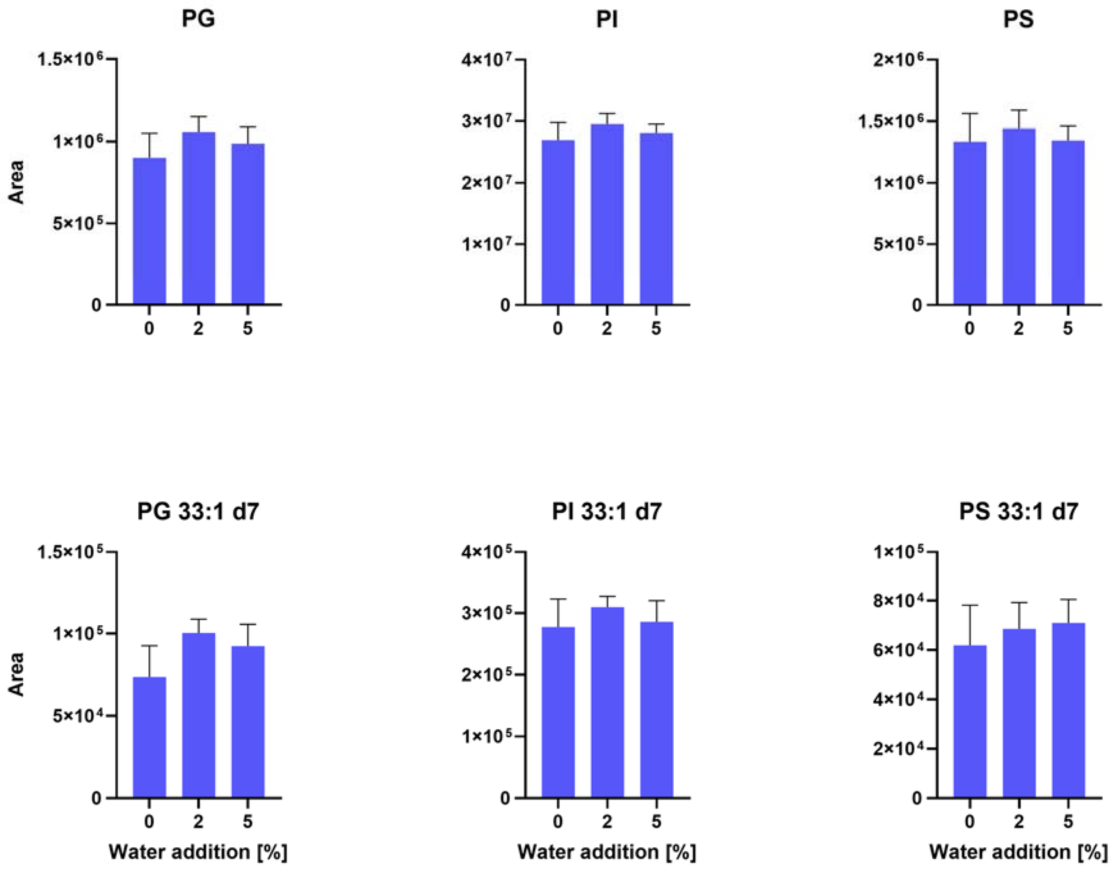

Figure S6. Histological specimens of normal (A1-A3) and fatty (B1-B3) pancreas, stained with hematoxylin and eosin (H&E), presented at various magnifications. Adapted from: Rugivarodom, M. et al., (2022). Fatty pancreas: linking pancreas pathophysiology to nonalcoholic fatty liver disease. *Journal of Clinical and Translational Hepatology*, 10(6), 1229.

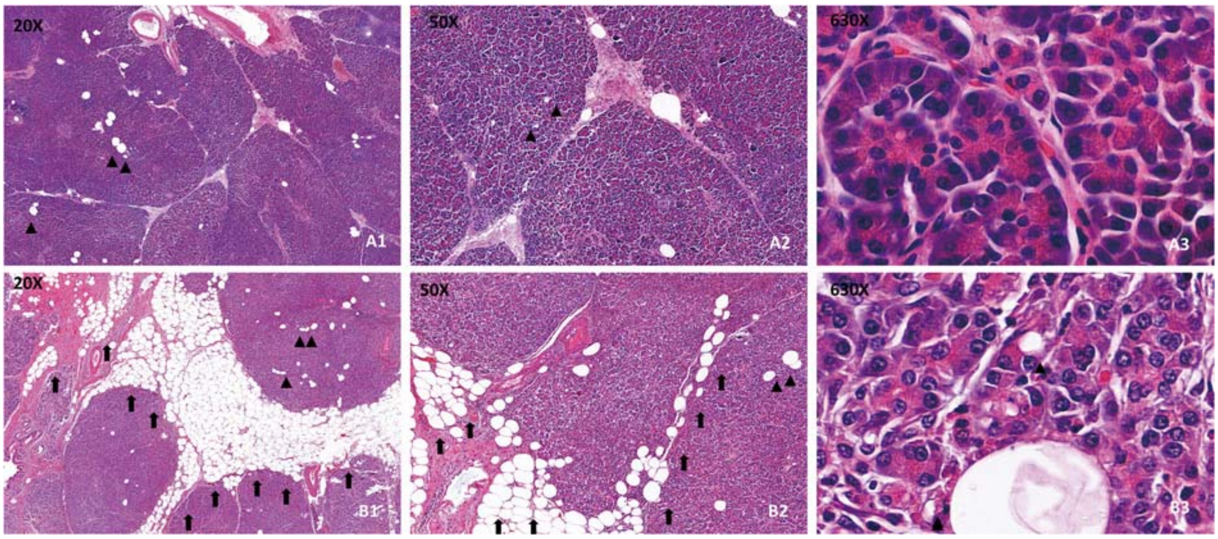

Figure S7. Concentrations of individual lipid species in pancreatic tissues of female (red dots) and male (blue squares) mice. Lines connect the concentration points of the same lipid species across both sexes. Lipid species with the highest and lowest concentrations within each lipid class are indicated. Results are expressed as means in nmol/mg of wet tissue.

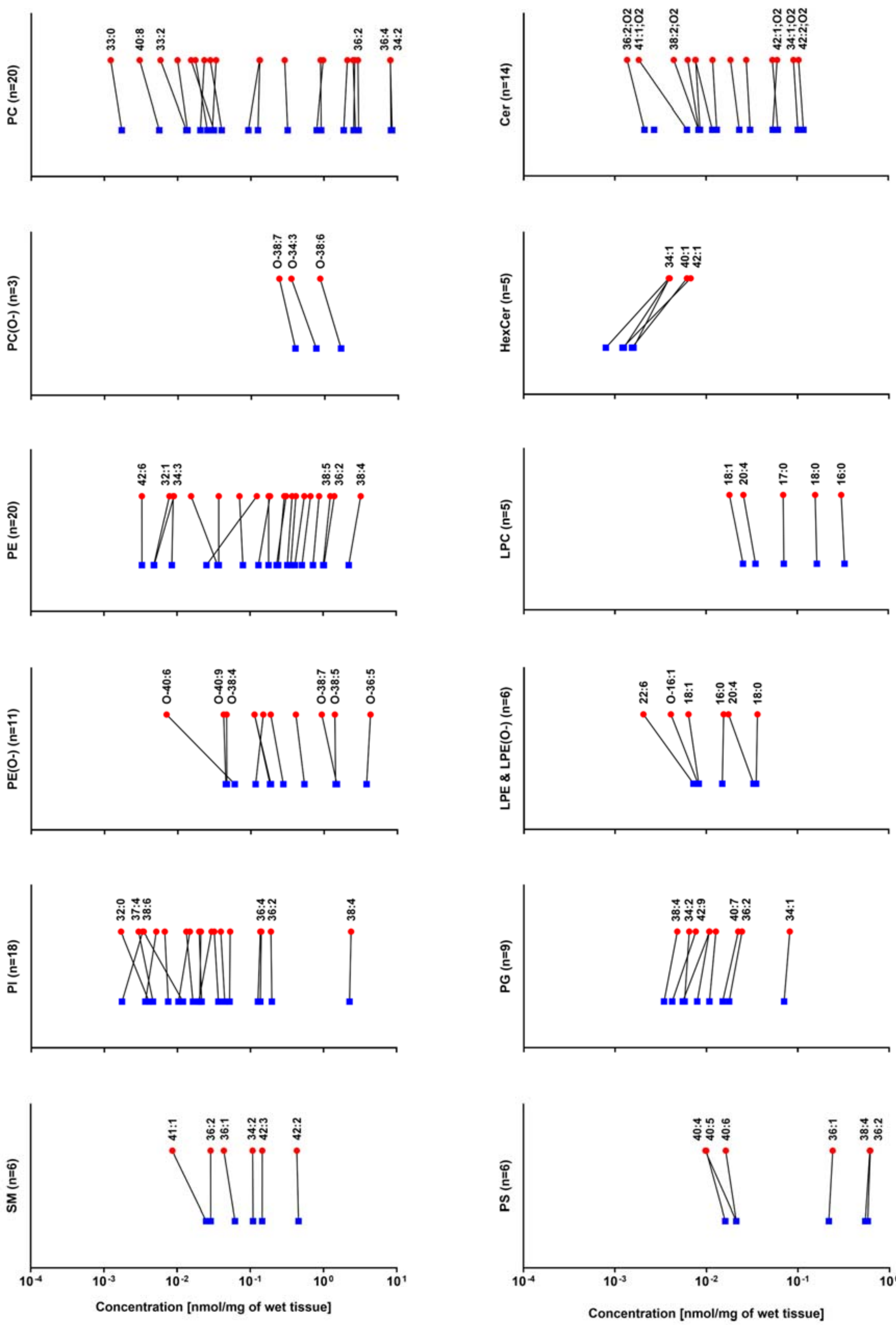

Supplement: Supplemental Data 3 [file mmc3.pdf]
